# Effects of Nitro-Oxidative Stress on Biomolecules: Part 1—Non-Reactive Molecular Dynamics Simulations

**DOI:** 10.3390/biom13091371

**Published:** 2023-09-11

**Authors:** Maryam Ghasemitarei, Tayebeh Ghorbi, Maksudbek Yusupov, Yuantao Zhang, Tong Zhao, Parisa Shali, Annemie Bogaerts

**Affiliations:** 1 Department of Physics, Sharif University of Technology, Tehran 14588-89694, Iran; 2 School of Engineering, New Uzbekistan University, Tashkent 100007, Uzbekistan; 3 School of Engineering, Central Asian University, Tashkent 111221, Uzbekistan; 4 Laboratory of Thermal Physics of Multiphase Systems, Arifov Institute of Ion-Plasma and Laser Technologies, Academy of Sciences of Uzbekistan, Tashkent 100125, Uzbekistan; 5 School of Electrical Engineering, Shandong University, Jinan 250061, China; 6 Research Unit Plasma Technology, Department of Applied Physics, Faculty of Engineering and Agriculture, Ghent University, 9000 Ghent, Belgium; 7 Research Group PLASMANT, Department of Chemistry, University of Antwerp, 2610 Antwerp, Belgium

**Keywords:** plasma medicine, reactive oxygen and nitrogen species, plasma nitro-oxidation, biomolecules, computer simulation, molecular dynamics

## Abstract

Plasma medicine, or the biomedical application of cold atmospheric plasma (CAP), is an expanding field within plasma research. CAP has demonstrated remarkable versatility in diverse biological applications, including cancer treatment, wound healing, microorganism inactivation, and skin disease therapy. However, the precise mechanisms underlying the effects of CAP remain incompletely understood. The therapeutic effects of CAP are largely attributed to the generation of reactive oxygen and nitrogen species (RONS), which play a crucial role in the biological responses induced by CAP. Specifically, RONS produced during CAP treatment have the ability to chemically modify cell membranes and membrane proteins, causing nitro-oxidative stress, thereby leading to changes in membrane permeability and disruption of cellular processes. To gain atomic-level insights into these interactions, non-reactive molecular dynamics (MD) simulations have emerged as a valuable tool. These simulations facilitate the examination of larger-scale system dynamics, including protein-protein and protein-membrane interactions. In this comprehensive review, we focus on the applications of non-reactive MD simulations in studying the effects of CAP on cellular components and interactions at the atomic level, providing a detailed overview of the potential of CAP in medicine. We also review the results of other MD studies that are not related to plasma medicine but explore the effects of nitro-oxidative stress on cellular components and are therefore important for a broader understanding of the underlying processes.

## 1. Introduction

Cold atmospheric plasma (CAP) is an ionized gas consisting of free radicals, ions, electrons, reactive neutral species, photons, and an electric field [1]. Recently, it has found numerous biological applications, including cancer treatment [2,3,4], wound healing [5,6], inactivation of microorganisms such as bacteria, fungi, and viruses [7,8,9], treatment of skin diseases [10,11], and blood coagulation [12]. Reactive oxygen and nitrogen species (ROS and RNS or RONS) derived from CAP play a crucial role in medical treatments [13]. Experimental studies have demonstrated that high levels of RONS as a result of CAP treatment can alter biological conditions at the cellular or tissue level, leading to improved and accelerated treatment outcomes [14,15].

Over the past few decades, CAP has emerged as a promising method for cancer treatment. Experimental studies have revealed that certain types of cancer cells are more susceptible to CAP treatment and oxidative damage compared to normal cells [16,17,18,19,20,21,22,23], although the reported selectivity depends on cell type and culture medium [24]. CAP exhibits therapeutic potential against various types of cancer [25], including hepatocellular carcinoma [26], lung carcinoma [27], breast cancer [28], melanoma [18], glioblastoma [29], pancreatic cancer [30], and head and neck cancer [31]. Intracellular RONS levels induced by CAP treatment in cancer therapies can cause irreversible damage to cellular components, particularly proteins, lipids, and DNA, ultimately leading to tumor cell death [32,33,34]. The extent of this damage varies depending on the CAP dose, and in some cases, it not only fails to destroy cells but also enhances cell proliferation, which is beneficial for wound healing [35]. Finding effective methods for healing chronic wounds, such as bedsores and diabetic wounds, is a major research focus due to their high prevalence, prolonged healing time, and associated costs [36]. Experimental studies have shown that wound reduction or closure can be achieved after short-term exposure to CAP ranging from 45 s to 2 min [37], without causing permanent damage to tissue or cells [38]. However, in cancer treatment, higher CAP doses are necessary to ensure cancer cell elimination [39,40].

Another application of CAP is the inactivation of microorganisms. RONS generated by CAP can damage the cell membrane, leading to leakage of intracellular biomolecules, reduced binding of oxidized membrane proteins to the cell, and DNA fragmentation through chain reactions [9,13].

Given the aforementioned explanations, various CAP devices have been designed for biological applications. CAP generally produces short-lived RONS, such as hydroxyl radical (^•^OH), atomic oxygen (O), ozone (O_3_), and nitric oxide (^•^NO), as well as long-lived RONS, including hydrogen peroxide (H_2_O_2_), nitrite (NO_2_^−^), nitrate (NO_3_^−^), and peroxynitrite (ONOO^−^) [41]. The concentration and types of RONS interacting with the target can be controlled depending on the CAP setup and duration of exposure to tissue, which is crucial for treating specific diseases [42].

During CAP treatment, cell membranes, and membrane proteins are the primary cellular components that can be chemically modified by RONS. The cell membrane plays a crucial role in separating the interior of the cell from the external environment and protecting the cell [43]. It also facilitates the transport of nutrients into the cell from the outside and removes toxic substances from the intracellular environment [44,45]. Structural changes induced by RONS in the membrane can disrupt its normal permeability to certain substances [46,47], including RONS produced by CAP [48,49,50]. Increased entry of RONS into the cell disrupts the balance between intracellular RONS levels and the antioxidant system, resulting in oxidative stress [51]. This oxidative stress can damage proteins, lipids, and DNA, affecting crucial interactions such as protein-protein, protein-lipid, and DNA-protein interactions, and disturbing the normal function of cell organelles [32,52,53,54,55,56,57]. The membrane also maintains membrane proteins, which have various functions in the cell, including membrane receptors, transport proteins, membrane enzymes, and cell adhesion molecules [58]. Any structural changes caused by RONS can disrupt the normal function of these membrane proteins, subsequently affecting intracellular reactions. Modifications to the membrane and membrane proteins, whether direct or indirect, can alter intracellular signaling pathways that are essential in bio-plasma therapy.

To gain a deeper understanding of the interactions between RONS and cellular components, which may not be fully explored using experimental techniques alone, computer simulations can be valuable [51]. Indeed, computer simulations provide insights into the effects of RONS on cellular and tissue components (such as proteins, lipids, and DNA) at the atomic scale, aiding in understanding the effectiveness of CAP in medical therapy [59,60] and facilitating the improvement of CAP devices to enhance their efficiency [61,62]. Among various computational simulations, reactive [63,64,65] and non-reactive [66,67,68,69] molecular dynamics (MD) simulations are powerful methods used to investigate the evolution of biological systems, considering their size and timescale. Reactive MD simulations are useful for studying the chemical reactions between RONS and biomolecules, involving the formation and breakage of chemical bonds. However, due to computational costs and resource limitations, the system size and timescale in reactive MD simulations must be limited. On the other hand, non-reactive MD simulations are suitable for studying the evolution of large systems and examining interactions between macromolecules, such as protein-protein, protein-membrane, and protein-DNA interactions. Although non-chemical interactions can be accurately studied using non-reactive MD simulations, the formation and breakage of chemical bonds cannot be explicitly modeled. Nevertheless, this method enables the investigation of system dynamics on a larger scale in terms of size and time [51,60]. This review paper discusses the application of non-reactive MD simulations in studying the changes occurring in two crucial components of cells, namely lipid membranes, and proteins, aiming to provide a broad overview of CAP in medicine.

## 2. Non-Reactive MD Simulations of Lipid Membranes

Cell membranes serve as a barrier between the interior of the cell and the external environment, and they play essential roles in regulating metabolism, intracellular signaling, and cell-to-cell communication [43]. Three main types of lipids are found in membranes, i.e., phospholipids, glycolipids, and sterols [44]. Among them, bilayer phospholipids are the primary components of eukaryotic membranes; they are structured with hydrophobic tails and hydrophilic head groups, allowing them to form a double layer of lipid molecules [70]. The integrity of membranes can be disrupted by various factors, including intracellular metabolic changes or extracellular stimuli like CAP interactions [48,50,71,72,73,74,75,76], specific drugs [77,78], and radiation [79]. These disruptions can result in damage to cellular functions and metabolism. CAP, as an extracellular stimulus, can modify the head groups or fatty acid chains of phospholipids through nitro-oxidation by RONS [48,71,76,80]. These modifications affect the properties of phospholipids and consequently impact their function as protectors of the cell against external invasions. The dynamic changes in membrane structure caused by lipid modifications induced by RONS can alter membrane properties and, subsequently, their function. Non-reactive MD simulations can be employed to study these modifications and their effects on membrane properties. In this section, we discuss the impact of membrane modifications by (CAP-generated) RONS on their properties and function using MD simulations. Specifically, we explore MD studies, both in the context of CAP treatment and beyond, i.e., nitro-oxidative stress in general. Indeed, the latter is also important as it provides insight into the broader processes of nitro-oxidative stress induced by (CAP-generated) RONS.

### 2.1. Effect of Nitro-Oxidation on Lipid Membrane Properties

**Figure 1:**
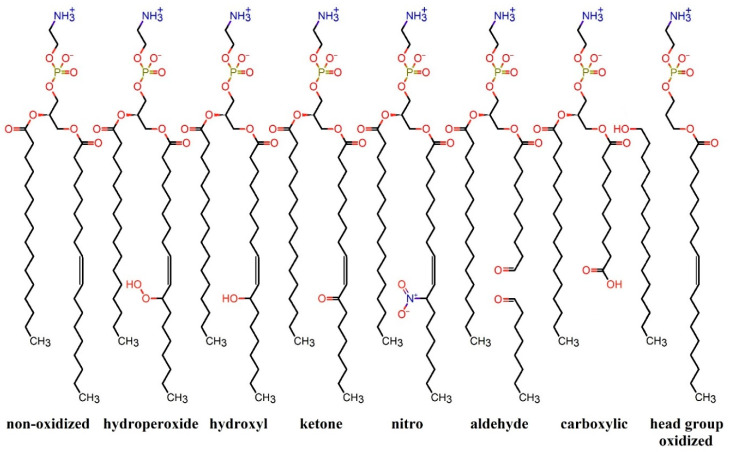
Schematic representation of the chemical modifications of the tail and head groups of phospholipids caused by (CAP-generated) RONS. The phospholipid POPC and its modified forms are shown as an example.

During CAP exposure, phospholipids and other membrane components can undergo nitro-oxidation through reactions with free radicals [71,81]. The phospholipid tails consist of two types of fatty acyl chains, i.e., saturated and *cis*-unsaturated, which can be located at positions one (*sn*-1) and two (*sn*-2), respectively. The double bonds present in unsaturated fatty acids (acyl chains) are more susceptible to the effects of (CAP-generated) RONS, resulting in the formation of hydroperoxide, hydroxyl, nitro, or acyl chain truncation [71,76]. Figure 1 presents different types of nitro-oxidative modifications on phospholipids caused by (CAP-generated) RONS, using palmitoyl-oleoyl-phosphatidylcholine (POPC) lipids as an example. Acyl chain cleavage involves replacing the hydrophobic unsaturated fatty acid at *sn*-2 with a shorter hydrophilic terminal functional group [82], such as aldehyde and carboxylic groups [83,84]. These terminal groups, which are highly hydrophilic, can destabilize membranes and subsequently alter their function, potentially leading to cell lysis, necrotic cell death, or programmed cell death [83,85,86], such as apoptosis [56,57] and ferroptosis [87]. The instability of nitro-oxidized membranes, which involves the nitro-oxidation of both the head and tail groups (see Figure 1), can be attributed to alterations in the membrane properties, in particular, fluidity, stiffness, and permeability to water, free radicals, and small molecules.

**Figure 2:**
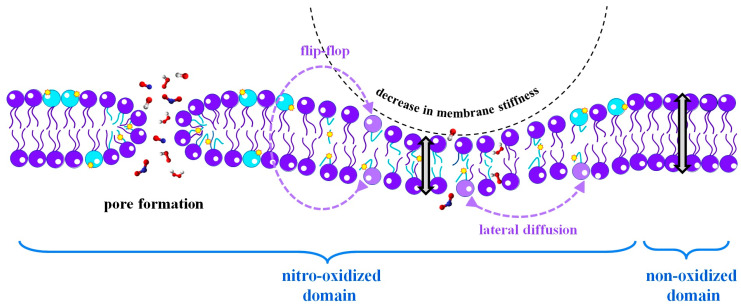
General alterations in membrane properties caused by nitro-oxidation. Non-oxidized and nitro-oxidized head and tail groups of phospholipids are shown in purple and cyan colors, respectively. The membrane thickness in both nitro-oxidized and non-oxidized domains is indicated by bidirectional vertical gray arrows. The phospholipids involved in lateral diffusion and flip-flop motion are shown in light purple color. The black dashed curvature indicates a decrease in membrane stiffness. RONS, represented by ball-and-stick models, are involved in the process of translocation across the lipid membrane, where pore formation and thickness reduction occur in the nitro-oxidized domain.

The nitro-oxidation of phospholipids typically results in alterations in membrane structures, which generally cause an increase in membrane fluidity and permeability, alongside a reduction in membrane stiffness. These changes are summarized and visually represented in Figure 2. Further elaboration on these properties is provided in the subsequent sections.

#### 2.1.1. Lipid Membrane Fluidity

Fluidity, defined as the relative mobility of individual lipid molecules, allows the membrane to adapt its shape and movement to different conditions and is related to their phospholipid tails [70]. It also affects the dynamics and function of membrane proteins and enables membrane lipids and proteins to diffuse from their site of synthesis to other areas of the cell where they are needed. Higher membrane fluidity allows for increased diffusion of membrane proteins through the membrane, leading to an increase in protein-protein interactions [87]. Membrane composition is a key factor in determining fluidity, which can be altered in pathological processes such as cancer formation [88,89,90,91] or in preparation for metastasis [92,93]. Modification of lipid composition by external methods, such as CAP exposure or chemotherapy, can modulate their fluidity, which has been an effective approach in cancer treatment [94]. Since cancer cells rapidly repair their membranes to maintain proliferation and evade apoptosis [95], membrane changes must be acute and sudden, which can be provided by CAP-induced oxidation. Oxidation of membrane lipids changes the lipid composition, affecting membrane fluidity and functionality [96]. Non-reactive MD simulation is a suitable method for studying the fluidity of oxidized membranes by analyzing the order parameters, lateral diffusion, and tilt angles of lipids [82,84]. Lateral diffusion refers to the movement of lipids within each leaflet of a membrane [97]. The order parameter indicates the degree of straightness or kink in the lipid tails [86] and is related to the tendency of lipid tails to align with each other, which correlates with the tilt angle—the angle between the C-H bond of the lipid tail and the normal axis of the bilayer. Tilting of unsaturated fatty acid tails results in altered lipid density, which can affect lipid fluidity [97]. This section considers the effect of nitro-oxidation of lipids by (CAP-generated) RONS on the fluidity of different types of lipid membranes.

Among the various types of phospholipids, those containing phosphocholine are the most abundant in the cell membrane [98,99]. Oxidation of these phospholipids by (CAP-generated) RONS can have a significant impact on membrane properties and cell function. Lipid oxidation primarily yields two main products: aldehyde and hydroperoxide groups [100]. These products are of particular interest in studying lipid oxidation using MD simulation. The aldehyde form of phospholipids is more stable and abundant compared to the hydroperoxide form [101,102]. Additionally, aldehydes diffuse faster across the membrane compared to hydroperoxides [100,103]. Wong-ekkabut et al. demonstrated that the oxidation of palmitoyl-linoleoyl-phosphatidylcholine (PLPC) bilayers with these two types of oxidation products increased the fluidity of the membrane [104]. Lipid oxidation caused the polar oxidized tails to bend towards the water surface and form hydrogen bonds with water molecules and the polar head groups. As the oxidation level increased, the lateral diffusion of the lipid tails increased, resulting in reduced membrane order and increased membrane fluidity [104]. Furthermore, the oxidation of POPC has been investigated for its effect on membrane fluidity. Beranova et al. conducted a study on POPC oxidation and found that the truncation of lipid tails, resulting in the formation of aldehyde and carboxylic groups, increased membrane fluidity [105]. Even at low levels of lipid oxidation, they observed an increase in lipid mobility and lateral diffusion of phospholipid head groups [105]. Van der Paal et al. [59] also studied the effect of shortening POPC lipid tails by (CAP-generated) RONS and the formation of aldehyde groups. They found that if a part of the separated tails remained in the membrane, it increased fluidity due to their high mobility within the membrane. These hydrophilic molecules, not attached elsewhere, tended to reach the water surface [59].

**Figure 3:**
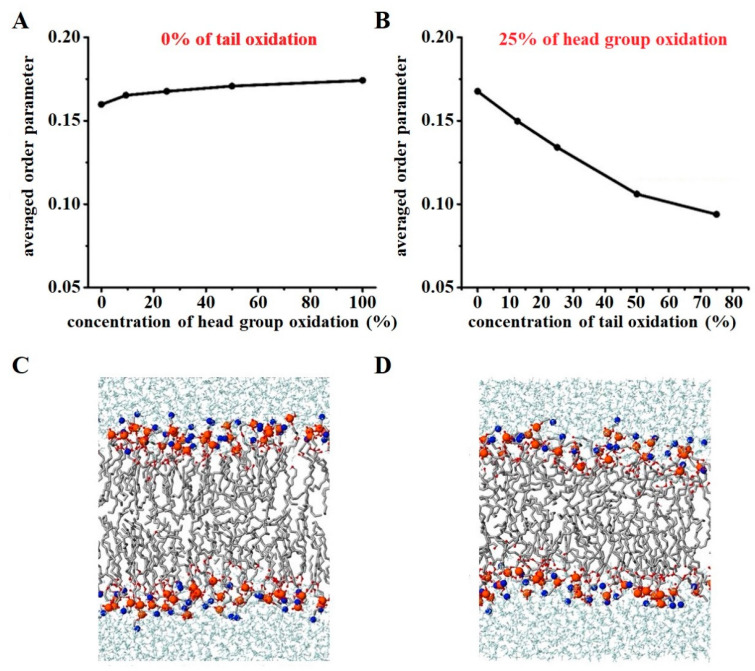
Average order parameter (i.e., a measure of the order of the lipid tails in the bilayer) as a function of the concentration of only head group oxidation (**A**) and tail group oxidation while keeping the oxidized head group at 25% (**B**). The order parameter is determined by $<3cos^{2}\theta -1>/2$, where *θ* is the angle between each C-H bond of the lipid tail and the bilayer normal (typically the z-axis). Note that an order parameter of 1 indicates that the lipid tails are perfectly aligned along the z-axis. Schematic pictures of membranes composed of only 50% head group oxidation (**C**) and 25% head group oxidation combined with 25% tail group oxidation (**D**). Reprinted/adapted with permission from Ref. [76]. 2017, The Authors.

In addition to tail group oxidation, the oxidation of phospholipid head groups can occur, albeit rarely, by (CAP-generated) RONS. Yusupov et al. [76] investigated the oxidation of both head and tail groups of dioleoyl-phosphatidylcholine (DOPC) and its impact on membrane properties through both experiments and MD simulations. They utilized the density functional-tight binding (DFTB) method to identify the products of ROS interacting with the head group of phospholipids. Based on both simulation and experimental results, they found that the main products observed are the oxidized head group (where one of the tails detaches) and the aldehyde form of the acyl chain, which is the dominant form of the oxidized tail group. The MD simulation results revealed that when only the DOPC head groups were oxidized, and their levels increased up to 100%, there was a slight increase in membrane order, leading to a minor decrease in membrane permeability (see Figure 3A). Interestingly, when both the head groups and lipid tails were oxidized simultaneously, the combined action generally resulted in a decrease in membrane order, leading to an increase in fluidity (see Figure 3B) [76]. Comparing Figure 3C (membrane containing 50% oxidized head group) to Figure 3D (membrane composed of 25% oxidized head and 25% tail groups), the disorder of phospholipids after oxidation of both head and tail groups becomes clearly evident.

In another study, Aceves-Luna et al. examined the effect of hydroperoxidation of phosphocholine-containing phospholipids, such as DOPC, on membrane fluidity [86]. Their findings indicated that higher levels of oxidation led to a reduction in membrane order and phospholipid packaging, thereby increasing membrane fluidity, consistent with previous studies [97,106,107]. They also observed membrane disruption even at low levels of oxidation due to significant tilting of hydroperoxide lipids [45,85,86].

In addition to the phospholipid composition, cholesterol plays a crucial role in regulating membrane fluidity [108]. Decreasing the level of cholesterol in the membrane, particularly in cancer cells during metastasis, increases membrane fluidity and enables cancer cells to penetrate blood vessels [92,93]. This highlights the importance of cholesterol in membrane fluidity and its utilization in the design of lipid drug carriers as regulators of membrane fluidity. Liposomes with different fluidity can selectively target different aggressive cancer cells [109]. Moreover, cholesterol-rich liposomes prevent drug leakage [110,111,112] under oxidative stress conditions due to their modulated fluidity [113]. MD simulations conducted by Schumann-Gillett et al. [106] demonstrated that a cholesterol-rich membrane composed of non-oxidized and oxidized POPC (containing truncated acyl tails to form carboxylic and aldehyde forms) generated by RONS cannot disrupt membrane bilayers because cholesterol protects the membrane. It increases membrane tail order, reduces lateral diffusion and tilt angle, and subsequently decreases fluidity [106]. These findings are consistent with the MD simulations performed by Khandelia et al. [114], which showed that the conical form of oxidized POPC complements cholesterol, resulting in the co-localization of oxidized POPC and cholesterol in the bilayers. This co-localization makes the membrane more compact, leading to reduced fluidity [114]. Building upon this study, Van der Paal et al. found that even if 100% of POPC is oxidized to the aldehyde form, a higher concentration of embedded cholesterol in the membrane can decrease fluidity due to the increased order of lipids, thus increasing membrane stability [59]. In addition to the oxidation of phospholipids, cholesterol can also undergo oxidation, which affects membrane fluidity [115]. The unsaturated double bond region of cholesterol is most easily oxidized by HOO^•^ [116]. Oxysterols, as oxidized forms of cholesterol, have pathogenic effects in cardiovascular diseases [117], diabetes [118], and degenerative disorders such as Alzheimer’s disease [119,120]. Therefore, studying their properties through MD simulations in bio-plasma therapy is important. Although oxysterols differ chemically only slightly from non-oxidized cholesterol, they cause significant changes in membrane properties [115,121]. Cholesterol can be oxidized from both its ring and tail groups, resulting in different alterations in membrane properties. Structural analysis of both types of oxidation revealed that oxysterols with oxidized tails have a similar effect on membrane fluidity as normal cholesterol. They increase the order of phospholipids, reduce lateral diffusion, and decrease membrane fluidity [115,122]. Tail-oxidized cholesterol mostly orients parallel to the bilayer normal in each leaflet, with its hydroxyl group positioned toward the head groups of phospholipids near the water interface. In contrast, oxysterol with an oxidized ring behaves somewhat differently than normal cholesterol [122]. It induces a greater tilt angle in membrane phospholipids and significantly disrupts membrane structure compared to tail-oxidized and normal cholesterols [115,122]. (CAP-generated) RONS primarily produce ring-oxidized cholesterol [115], which can reduce membrane fluidity. The lower presence of cholesterol in the membrane of cancer cells leads to increased membrane fluidity during membrane oxidation and subsequently increases the probability of membrane disruption [92,93].

However, a study on the combination of ring-oxidized cholesterol and hydroperoxidation of POPC revealed that membrane fluidity does not change significantly [123]. This lack of change is attributed to the –OOH groups of hydroperoxidized POPC and oxidized cholesterol remaining closely together and not moving toward the phospholipid head groups [123]. These findings are important in the treatment of cancer cells using (CAP-generated) RONS.

#### 2.1.2. Lipid Membrane Stiffness

Stiffness, or the resistance to elastic deformation, is an important physical characteristic of the cell membrane and is measured by the elastic modulus. In the context of cancer studies, the decrease in membrane stiffness is associated with the transformation, malignancy, and metastasis of cancer cells. This decrease in stiffness is attributed to both the cytoskeletal network and the cell membrane [124]. Metastatic cancer cells typically exhibit lower levels of cholesterol in their membranes, resulting in a softer membrane that can change shape and deform more easily, thereby increasing their invasive capacity [125,126]. On the other hand, multidrug-resistant cancer cell membranes often contain higher levels of cholesterol, which makes the membrane more rigid and less permeable to drugs [127]. Consequently, new strategies are being explored to design lipid drug carriers specifically for multidrug-resistant cancer cells by manipulating different lipid types and cholesterol levels [128].

**Figure 4:**
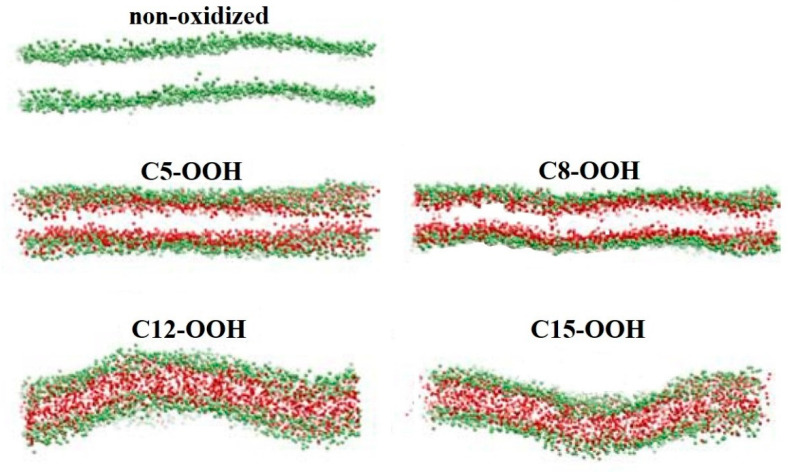
Schematic representation of a membrane composed of non-oxidized SAPE and 100% oxidized SAPE at various oxidation sites. The SAPE head groups are illustrated in green, while the oxidized domains are highlighted in red. Reprinted/adapted with permission from Ref. [130]. 2021, Elsevier.

Therefore, novel approaches, such as CAP exposure, are needed to facilitate drug delivery to multidrug-resistant cancer cells by targeting changes in lipid and cholesterol compositions. Generally, there is an inverse relationship between membrane stiffness and fluidity. Lipid oxidation increases fluidity and subsequently decreases membrane stiffness due to the lower elastic and bending modulus of the oxidized membrane region. In the previous section, we discussed how lipid oxidation increases membrane fluidity. In this context, we highlight an anomalous experimental study by Dobretsov et al. that demonstrated an increase in membrane stiffness despite increased lipid oxidation [129]. In their study, lipid oxidation was induced in liposomes composed of a mixture of phosphatidylcholine (PC) and phosphatidylethanolamine (PE) components, and it was observed that the liposome membrane became stiffer with lower fluidity [129]. Following this study, Chng et al. employed MD simulations to investigate which type of oxidation contributes to increased membrane stiffness [130]. Their system comprised phospholipids, specifically stearoyl-arachidonoyl-PE (SAPE) and stearoyl-arachidonoyl-PC (SAPC), which contain polyunsaturated fatty acids with four double bonds. They examined lipid peroxidation at four specific sites, two near the head group and two close to the bilayer interior. By calculating the elastic modulus, they observed that while membrane fluidity increased overall, oxidation near the phospholipid head group (C5-OOH and C8-OOH) led to increased membrane stiffness and order, whereas oxidation near the bilayer interior (C12-OOH and C18-OOH) resulted in membrane softening and disruption (see Figure 4) [130].

Hence, the site of oxidation in the acyl chains of phospholipids with multiple oxidation sites plays a crucial role in membrane stiffness, which should be considered during CAP exposure, as sites near the head group are more prone to oxidation compared to other sites.

#### 2.1.3. Lipid Membrane Permeability

The permeability of the lipid membrane is a crucial property that determines the passage of solutes through the membrane, allowing access to the inside of the cell and regulating cell homeostasis and the cell cycle [131]. Small metabolite molecules essential for cell survival can be transported across the membrane through transmembrane proteins (actively) or directly through the lipid bilayer (passively). During CAP exposure, changes in the lipid composition of the membrane can occur, affecting its permeability, in particular, the passive uptake of metabolite molecules [132]. One important factor related to permeability is the membrane thickness, which can be easily analyzed. A thinner membrane is more permeable to water, and different states of the membrane, such as the liquid-disordered state, liquid-ordered state, and gel state, are characterized based on their thickness [133]. Oxidation of phospholipid tails by (CAP-generated) RONS, which occurs due to increased exposure of the lipid tails, leads to a decrease in membrane thickness and an increase in water permeability [72,86,134]. Another parameter associated with membrane permeability is the average surface area per lipid. A higher value indicates increased permeability and greater access to the intracellular environment from the outside [72,86,134,135]. In oxidized membranes, the bending of the oxidized polar groups toward the water interface often leads to an increase in the average surface area per lipid, resulting in enhanced permeability [45,59,75,81,87,104,136]. MD simulations performed on oxidized lipids, such as oxidized DOPC [45,86,137], oxidized POPC [45], and oxidized PLPC [104], have demonstrated that water density near the head groups increases, shifting the lipid density towards the center of the bilayer. Additionally, the average surface area per lipid increases, leading to a decrease in membrane thickness and ultimately an increase in permeability [75,138].

Pore Formation in the Membrane due to Lipid Oxidation

Higher permeability of membranes can lead to the formation of pores, allowing water and other solutes to pass more easily through the membrane [139]. Pore formation occurs when the head groups of both leaflets reorient towards the center of the bilayer, interacting with each other and creating a stable pore for solute transport [72]. Pore formation can occur spontaneously through cellular mechanisms or as a result of external stimuli such as electric fields [140,141,142], mechanical forces [141], ionic gradients [143], surfactants [144], certain peptides [145,146], and oxidation of phospholipids by (CAP-generated) RONS [59].

**Figure 5:**
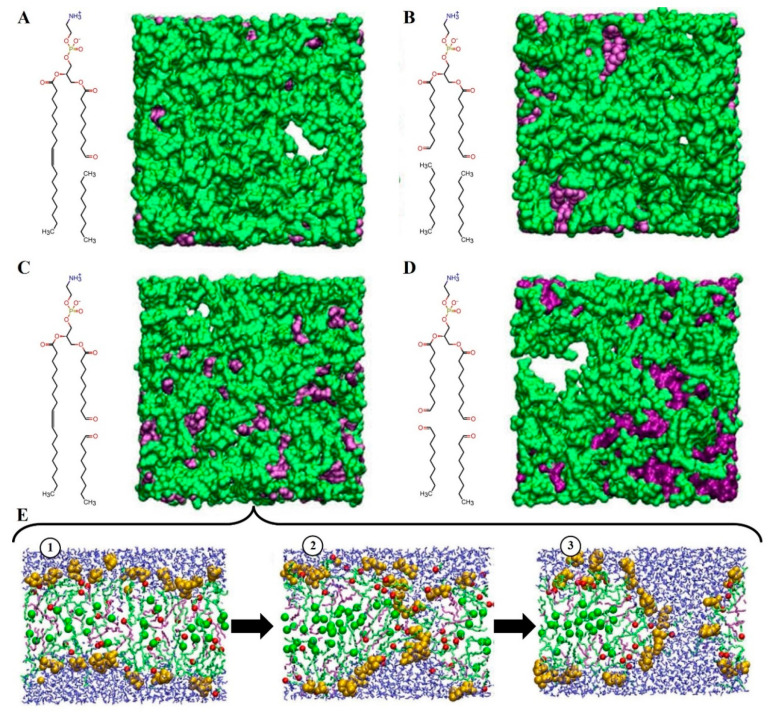
(**A**–**D**) Chemical structure of the oxidized DOPC lipid on the left and the top view of a membrane composed of these lipids on the right. In all cases, the DOPC lipids are oxidized in the aldehyde form at the sn-1 position (**A**,**C**) or at both sn-1 and sn-2 positions (**B**,**D**). The separated short chains resulting from cleavage contain either hydrophobic methyl groups (**A**,**B**) or hydrophilic aldehyde groups (**C**,**D**) at the cleavage sites. (**E**) Representation of the three stages of pore formation during the MD simulation of the membrane composed of oxidized DOPC lipids, as shown in (**C**). Reprinted/adapted with permission from Ref. [147]. 2010, Elsevier.

In oxidized membranes, pore formation is a result of increased distances between the lipid head groups due to the increase in the surface area of each lipid, leading to the formation of relatively large water-filled pores [46,75,139,147]. MD simulations have been used to study pore formation in oxidized membranes, providing valuable insights into the properties of oxidized membranes relevant to CAP therapy in various cell types with different membrane compositions [59,75]. The effect of oxidation of PC lipids on pore formation in membranes has been investigated. For instance, Wong-Ekkabut et al. conducted an MD simulation study on oxidized PLPC and reported that membrane pores were formed after 5% oxidation of PLPC (in the aldehyde and hydroperoxide forms), and these pores became more stable at higher oxidation levels [104]. However, another study on PLPC hydroperoxidation found contradictory results, showing that membrane pores were not formed after 5% lipid oxidation [139]. In particular, Boonnoy et al. performed MD simulations on oxidized PLPC and palmitoyl-decanoyl-PC (PDPC) in the aldehyde and hydroperoxidized forms. They found that water defects and stable pore formation only occurred in the aldehyde forms after 50% lipid oxidation. They also observed that more than 50% lipid oxidation led to the formation of unstable pores, membrane rupture, and eventual micellization [139]. These findings were supported by Oliveira et al., who studied the oxidation of POPC to the aldehyde form and found that pore formation was not observed up to 25% lipid oxidation, but 100% oxidation of POPC resulted in pore formation [74]. Beranova et al. investigated the cleavage and oxidation of POPC to the aldehyde and carboxylic forms, and they found that water molecules penetrated more deeply into POPC oxidized to the aldehyde form compared to the carboxylic form [105]. Volinsky et al. also indicated that the lipid flip-flop rate increased in a POPC bilayer membrane containing these two types of oxidation up to 20%, leading to the formation of more pores [148]. The aldehyde form of oxidized phospholipids has been extensively studied and considered the most stable form in many investigations [101,102]. However, the influence of the separated short chains remaining in the membrane on its permeability has not been studied there. With this in mind, Cwiklik et al. studied the permeability of a homogenous membrane containing 100% oxidized DOPC lipids in the aldehyde form along with separated short chains resulting from cleavage [147]. They considered two possible forms for the separated short chains. One of the forms involved a hydrophilic aldehyde, while the other had a hydrophobic methyl group at the cleavage site. Both of these forms were considered to be formed at the *sn*-1 position, as well as at both *sn*-1 and *sn*-2 positions. Figure 5A–D shows these forms of separated short chains (left) and equilibrated membranes composed of 100% aldehyde-oxidized DOPC lipids (right) that incorporate these short chains. As is clear, pore formation can occur in these membranes, as illustrated by the three steps depicted in Figure 5E. In general, the authors found that, in contrast to a separated short hydrophilic chain, if the remaining fragment was hydrophobic, the thickness of the oxidized membrane increased, resulting in a decrease in permeability. The hydrophobic fragments tended to remain inside the membrane, preventing membrane shrinkage and entanglement of the remaining acyl chains. However, the overall membrane integrity was maintained for both hydrophobic and hydrophilic short-chain forms when one of the two acyl chains of DOPC was oxidized. In contrast, when all acyl chains were oxidized, the oxidized DOPC membrane bilayer disintegrated, resulting in micellization [147].

Along with the study, Lis et al. investigated pore formation in membranes with different levels of oxidized DOPC, considering the aldehyde form of separated short-chains [46]. They found that moderate membrane oxidation allowed for poor water permeation, with only single molecules diffusing across the membrane. As the oxidation level increased, water clusters were able to transfer across the membrane, and transient chains of water molecules formed. Finally, at the highest oxidation level, membrane water pores were formed [46]. Therefore, membrane damage resulting from oxidation depends on the level of oxidation and the chemical properties of the oxidized membrane compounds.

In addition to phospholipid types and their various oxidations, other components, such as cholesterol, also play a significant role in membrane permeability and pore formation. Cholesterol molecules, which reduce the fluidity of the oxidized membrane, can act as an adhesive in the membrane. By organizing and compacting the membrane, they decrease the area per lipid and increase membrane thickness, ultimately reducing membrane permeability and pore formation [115,121]. MD simulations have been used to study membrane permeability and pore formation in cholesterol-rich membranes composed of non-oxidized and oxidized phospholipids. Different studies on various types of oxidized membranes have shown that increasing the cholesterol level generally decreases pore formation [114,149,150,151], and a higher level of lipid oxidation is required to observe membrane pores [152]. However, a study on a homogenous membrane containing 100% POPC oxidized to the aldehyde form showed that adding only 11% cholesterol protected the membrane from pore formation [59]. Cholesterol’s protective role is attributed to its ability to create hydrogen bonds with oxidized phospholipids, facilitate rapid flip-flop, and reduce differential density stress caused by the asymmetric shape of the oxidized membrane leaflet [59]. Boonnoy et al. investigated the flip-flop rate of cholesterol in oxidized PLPC membranes with aldehyde and hydroperoxide forms [151]. Their MD simulation results, combined with umbrella sampling (US) simulations, showed that the energy barriers for cholesterol translocation from one leaflet to the other were about 20 kJ/mol and 6 kJ/mol for the hydroperoxide and aldehyde forms of PLPC, respectively. They observed that only cholesterol flip-flop was observed in membranes containing the aldehyde form, and the rate of flip-flop decreased with increased membrane cholesterol levels. Therefore, membranes containing the aldehyde form were more protected against stress due to high cholesterol flip-flop rates compared to hydroperoxidized membranes [151]. However, this study did not consider the flip-flop rate of the aldehyde form of PLPC, which could still induce differential density stress.

Besides, experimental studies and MD simulations on oxidized membranes have demonstrated that adding cholesterol to the membrane can reversibly repair fragile oxidized membrane pores [114,149]. Although cholesterol-rich membranes are generally more resistant to pore formation, it is important to note that during exposure to CAP, cholesterol can also undergo oxidation. The effect of oxidized cholesterol (or oxysterol) on membrane permeability depends on the type of cholesterol oxidation. According to research by Kulig et al. [115], both tail-oxidized cholesterol and ring-oxidized cholesterol result in a decrease in membrane thickness. However, the reduction in thickness is significantly higher in membranes containing tail-oxidized cholesterol. The effect of ring-oxidized cholesterol on membrane thickness is similar to that of cholesterol itself. Generally, the arrangement of lipid tails, the average area per lipid, membrane thickness, and the tilt of cholesterol and phospholipid tails are interconnected parameters. It is expected that an increase in the area per lipid and higher tilt of tails would lead to a reduction in membrane thickness and lipid tail order. However, in the case of oxysterols, where the cholesterol tail is oxidized, the mentioned dependencies between the parameters are not observed, and membrane thickness is reduced. This could be explained by the tendency of oxysterols with the oxidized form of their tail to move from one leaflet to another, facilitated by the lowered energy barrier for flip-flop, resulting in local thinning of the membrane [115]. This thinning of the membrane may facilitate pore formation. The flip-flop rate of ring- and tail-oxidized cholesterol was investigated in oxidized neuronal membranes by Wilson et al. [122]. They observed that tail-oxidized cholesterol flip-flopped faster than ring-oxidized cholesterol, which could explain the reason for the thinning of oxidized membranes containing tail-oxidized cholesterol [123]. Furthermore, the simultaneous oxidation of phospholipids and ring-oxidized cholesterol was considered in MD simulations. The results showed that the membrane composed of oxidized phospholipids and cholesterol had the same thickness and membrane permeability as the pure and intact membrane [123].

Membrane Pore Formation Due to Lipid Oxidation in Combination with External Stress or Electric Field

Rupture of the cell membrane is experimentally considered an indicator of necrotic cell death [153,154]. It occurs when the tension or strain on the cell membrane surpasses critical values, which depend on the membrane composition and the intracellular and extracellular environment [155]. The speed at which stretching occurs is a crucial factor that determines whether the membrane has sufficient time to repair and protect itself from rupture [156].

Oliveira et al. conducted a study using MD simulations to investigate the effect of mechanical stress on four types of homogeneous membranes: 100% POPC and three types of oxidized POPC (hydroperoxidation, alcohol, and ketone forms) [72]. They observed that stretching the membranes caused them to become thinner until the areal strain reached a critical value, at which point pore formation began. Areal strain refers to the ratio of surface change to the initial membrane surface area, and its critical value is lower for oxidized POPC membranes compared to non-oxidized ones. The speed of stretching is an important factor that influences the onset of pore formation. Generally, under slower stretching speeds, larger strain and more time are required for pores to appear compared to higher-speed stretching. The results also indicated that pore formation in the oxidized membrane, particularly in the hydroperoxidized and alcohol forms of POPC, could occur with lower strain compared to non-oxidized POPC and POPC oxidized to the ketone form. Additionally, the researchers combined POPC and hydroperoxidized POPC bilayers (i.e., a membrane with two domains) to study the effect of mechanical stress on this heterogeneous membrane. They found that although the two domains were not mixed, this membrane was more susceptible to pore formation than the non-oxidized POPC membrane and behaved similarly to the homogeneous hydroperoxidized POPC membrane. Furthermore, there was no preference for pore formation between the interface or bulk of the two-domain membrane [72]. Therefore, mechanical stress can facilitate pore formation and membrane leakage, especially in oxidized membranes. Moreover, if the stretching speed is higher, the membrane lacks sufficient time for repair, increasing the likelihood of pore formation and membrane rupture.

In addition to mechanical stress, an electric field can increase membrane permeability and facilitate pore formation, particularly in oxidized membranes [75,140,142,157,158]. Certain CAP sources generate strong electric fields, in addition to RONS [62]. Thus, studying the impact of electric fields on pore formation in oxidized membranes is crucial in CAP therapy. Vernier et al. investigated this effect on membranes containing non-oxidized and oxidized PLPC (aldehyde and hydroperoxidized forms) [158]. They found that the external electric field reduced the time required for pore formation in oxidized membranes, with a shorter time observed for PLPC oxidized to the aldehyde form compared to other oxidized forms. Furthermore, an increase in the magnitude of the electric field in the 50% oxidized PLPC membrane resulted in decreased time for electro-pore formation, particularly for the aldehyde form [158]. Along similar lines, Yusupov et al. demonstrated that the electrical effect can facilitate pore formation in membranes composed of non-oxidized DOPC and DOPC oxidized to the aldehyde and hydroperoxidized forms, and their results aligned with the previous study [75]. Besides, Wang et al. found that both nanosecond pulsed electric field and picosecond pulsed electric field may lead to membrane electroporation, and the electroporation time decreases exponentially with the increase of electric field intensity [159]. Therefore, higher oxidation levels and electric fields induced by CAP can elevate the probability of membrane pore formation, and subsequent membrane rupture (particularly in cell membranes), and ultimately lead to necrotic cell death.

### 2.2. Effect of Membrane Nitro-Oxidation on Plasma Therapy

Based on the previous sections, lipid oxidation in membranes can play a significant role in CAP therapy due to the increased vulnerability of the membrane to penetration and structural changes. Lipid oxidation generally renders the membrane softer, with higher fluidity and increased permeability to drugs and essential metabolite molecules in the treatment of certain diseases [85,132,160,161,162,163,164,165]. Additionally, the membrane acts as the initial barrier to the penetration of RONS into the cell during exposure to CAP. Oxidation of the membrane and the resulting increase in permeability facilitates the penetration of RONS, which is beneficial for inducing intracellular oxidative stress and altering cell signaling [51,75,81,166,167]. In this section, we will discuss some applications of CAP in therapy, considering oxidized membranes.

When exposed to the skin, RONS generated by CAP can interact with the stratum corneum (SC), the outermost layer of the skin that contains ceramides (CER), free fatty acids (FFAs), and cholesterol [168,169,170]. Therefore, studying SC oxidation and its effect on the skin membrane and the subsequent passage of RONS through it could be valuable in bio-plasma therapy. Van der Paal et al. proposed that the oxidation of CERs by CAP-generated RONS leads to cross-linking between CERs [49]. Their MD simulation results demonstrated that this cross-linking leads to the creation of pores in the membrane, and the size of these pores expands with increasing oxidation level and cross-linking between CERs. As a result, it facilitates the passage of drug molecules and CAP-generated RONS through the skin membrane [49]. Other studies on SC cholesterol oxidation have shown that ring-oxidized cholesterol increases the surface area per lipid and reduces membrane thickness, thereby increasing membrane permeability. Structural analysis of membranes containing ring-oxidized cholesterol in the hydroperoxidized form revealed lateral expansion of the membrane due to the movement of the oxidized group toward the water surface and the tilting of the oxidized cholesterol tail. The free energy of transport of major CAP-generated RONS through non-oxidized and oxidized SC membranes, obtained through MD simulations, demonstrated that the free energy barrier for RONS passage through the SC membrane generally decreases with increasing cholesterol oxidation level [171,172]. Among all the investigated RONS, hydrophobic RONS (e.g., ^•^NO, ^•^NO_2_, O_2_, O_3_, and N_2_O_4_) could cross the membrane more easily, with a lower free energy barrier, compared to hydrophilic RONS (e.g., H_2_O_2_, ^•^OH, HOO^•^, HNO_2_, and HNO_3_) and ions (e.g., NO_2_^−^ and NO_3_^−^) [171]. Experimental results align with MD simulations, indicating that RONS permeability through the SC membrane increases when the skin is moist, and the membrane is oxidized [171]. However, short-lived RONS were found to have limited penetration into the SC layer due to their highly reactive nature, resulting in half-lives of a few nanoseconds [173]. Hence, the penetration of RONS into the skin membrane depends on its composition, which is essential for the specific functions of skin cells.

**Figure 6:**
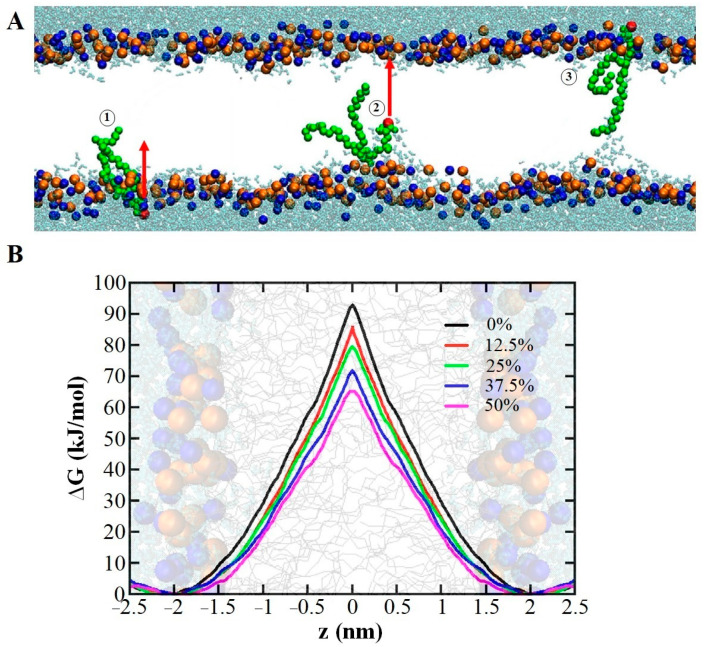
(**A**) Schematic illustration of three steps of the PS flip-flop motion from the inner leaflet to the outer leaflet. The red arrow indicates the direction of pulling PS head group that induces the flip-flop movement. (**B**) Symmetrized free energy profiles for the passage of PS across the POPC membrane with different hydroperoxidation levels. Reprinted/adapted with permission from Ref. [178]. 2017, John Wiley and Sons.

One of the distinguishing characteristics between cancer cells and normal cells is their membrane composition, which renders cancer cells more susceptible to CAP treatment and oxidative damage [49,59], ultimately leading to apoptotic cell death [174]. Phosphatidylserine (PS) is one of the markers of apoptotic cell death and is typically found in the inner leaflet of the cell membrane [175]. During the activation of apoptotic pathways, PS undergoes a flip-flop mechanism, moving from the inner to the outer leaflet, thereby acting as an “eat me” signal for lymphocyte cells [176]. Increasing the rate of PS flip-flop can induce apoptosis [176]. As mentioned earlier, CAP-induced oxidation of the membranes increases their permeability, facilitating the flip-flopping of PS between the membrane leaflets. Additionally, membrane defects caused by the oxidation of polyunsaturated phospholipids provide energetically favorable pathways for the flip-flopping of polar groups of phospholipids (e.g., PS) across the membrane [177]. The energy barrier for PS flip-flop in membranes containing unsaturated POPC oxidized to hydroperoxide and aldehyde forms was investigated by Razzokov et al. [178] and Volinsky et al. [148], respectively, using MD simulations. The study on the effect of POPC hydroperoxidation on PS flip-flop demonstrated that as the oxidation level increased from 0% to 50%, the energy barrier for PS flip-flop decreased by approximately 30%, from 90 kJ/mol to 60 kJ/mol, thereby increasing the rate of PS flip-flop (see Figure 6). Moreover, during the transition of PS from one leaflet to another, a hydrophilic narrow pore was formed [178].

In the case of POPC oxidized to the aldehyde form, the MD results showed that the flip-flop rate of PS increased with increasing oxidation level. Oxidation of only 20% of POPC resulted in an energy barrier for PS flip-flop of 80 kJ/mol, which was approximately 20 kJ/mol lower than the energy barrier for non-oxidized POPC [148]. Thus, increasing the rate of PS flip-flop, as an indicator of apoptotic cell death due to membrane oxidation by CAP-generated RONS, could be beneficial in cancer therapy.

Increased membrane permeability through oxidation can also facilitate the absorption of cancer drugs by the membrane. Melittin is one such drug that exhibits inhibitory effects on the proliferation of various types of cancer. Despite its efficacy against various cancers [179], its clinical application is limited due to its toxicity at high doses [180]. Therefore, enhancing its uptake can enhance its effectiveness at lower doses and reduce its side effects. Shaw et al. conducted MD simulations to compare the absorption of melittin from oxidized and non-oxidized membranes [164]. Their free energy results obtained through US simulations showed that it is much easier for melittin to pass through a membrane composed of 50% POPC oxidized to the aldehyde form compared to its passage through a non-oxidized membrane. Consequently, due to membrane oxidation, the likelihood of melittin penetrating into the cell increases. Thus, lower doses of melittin, which were previously cytotoxic, can be utilized in cancer treatment [164].

In addition to the impact of CAP on membrane oxidation and its alteration of membrane properties, which are crucial in CAP therapy, the penetration rate of different types of RONS into the membrane becomes significant. The permeation of RONS through the membrane affects changes in intracellular components, such as proteins, which play a vital role in cell signaling. The results on the free energy of penetration of different types of RONS through non-oxidized and nitro-oxidized membranes obtained using US simulations are discussed below. The properties of phospholipids, including their polarity, type of lipid tails, size of head groups, and type of oxidation, as well as the size and polarity of RONS, have an impact on RONS penetration [50,70,89,123,181,182,183,184].

To investigate the influence of head and tail groups of membrane phospholipids on the permeation of ROS, Van der Paal et al. conducted a comprehensive study utilizing both experiments and MD simulations [50]. The particular focus was on H_2_O_2_, a long-lived, polar, and the largest ROS produced by CAP. The study examined membranes with different lipid compositions, namely DOPC, dipalmitoyl-PC (DPPC), and dipalmitoyl-PE (DPPE). DOPC and DPPC have similar head groups but differ in their lipid tails, with DOPC having unsaturated tails and DPPC having saturated tails. On the other hand, DPPC and DPPE possess similar tail groups but differ in their head groups. Hence, an investigation was conducted to examine the translocation of H_2_O_2_ across membranes of varying lipid compositions. The focus was on three factors: (i) the saturation degree of lipid tails, (ii) the type of lipid head group, and (iii) the proportion of membrane cholesterol [50]. The aim was to gain insights into how the interaction between phospholipids and cholesterol could affect the response of healthy and cancerous cell membranes to ROS derived from plasma. The experimental findings revealed that an increase in DPPE concentration within the DPPC or DOPC vesicles led to tighter lipid packing, resulting in a decrease in lipid area. Consequently, the passive diffusion of ROS would be impeded in both systems as the DPPE fraction increased. This observation was also supported by the US simulations of DPPC vesicles, which demonstrated an elevated free energy barrier for H_2_O_2_ permeation with increasing DPPE content [50]. However, contrary to the simulation results, the experimental data for DOPC vesicles indicated enhanced permeability as the DPPE fraction increased. This disparity can be explained by the potential formation of lipid rafts enriched in DPPE and cholesterol when the DPPE content is increased. As a result, other regions of the membrane may become enriched in DOPC, making them susceptible to pore formation due to lipid oxidation, thereby facilitating increased ingress of ROS [50]. This phenomenon accounts for the heightened ROS penetration observed in DOPC vesicles with increased DPPE content. In DPPC vesicles, no such rafts are formed because both DPPC and DPPE possess identical aliphatic lipid tails and exhibit equally strong interactions with cholesterol. Overall, the experimental and simulation results highlight the involvement of multifactorial chemical and physical processes, including lipid oxidation, lipid packing, and lipid raft formation [50]. This study has potential implications for the development of therapies centered around CAP, which targets the cell membrane and oxidative stress response in cells.

In addition to ROS, RNS produced by CAP can also penetrate the membrane. Razzokov et al. compared the permeation of ROS (O_2_, O_3_, ^•^OH, HOO^•^, and H_2_O_2_) and RNS (^•^NO, ^•^NO_2_, N_2_O_4_) through non-oxidized and oxidized membranes containing non-oxidized DOPC and 50% oxidized DOPC in the aldehyde form [81]. The free energy profiles of RONS permeation, obtained through US simulations, demonstrated significantly lower energy barriers for all RNS, O_2_, and O_3_ (hydrophobic species) with values ranging from 6 to 12 kJ/mol for non-oxidized membranes and 5 to 7 kJ/mol for oxidized membranes, compared to hydrophilic species such as ^•^OH, HOO^•^, and H_2_O_2_ (with energy barriers ranging from 20 to 35 kJ/mol for non-oxidized membranes and 15 to 25 kJ/mol for oxidized membranes). Among the hydrophilic species, H_2_O_2_ exhibited greater difficulty in permeating the membrane due to its size and its ability to form more hydrogen bonds with water [81]. Although H_2_O_2_ and other hydrophilic species cannot easily cross the membrane, they can pass through membrane pores formed due to membrane oxidation, the combination of membrane oxidation and a strong electric field generated by CAP, or be absorbed by membrane proteins such as aquaporins (AQPs) [80]. The importance of the type of lipid oxidation on ROS (i.e., ^•^OH, HOO^•^, H_2_O_2_, and O_2_) penetration across the membrane was investigated by Yusupov et al. [75] using US simulations. Their systems included non-oxidized DOPC, 50% oxidized DOPC in the aldehyde form, and hydroperoxidized DOPC, which are major products of phospholipid oxidation. The results indicated that after membrane oxidation, the free energy barrier for ROS passage through the membrane decreased, especially for the oxidized membrane in the aldehyde form, which exhibited significantly higher permeability than the hydroperoxidized membrane [75]. Van der Paal et al. [152] observed that the incorporation of cholesterol (from 0–50%) into the oxidized membranes increased the free energy barrier for ROS permeation compared to cholesterol-free membranes. This can be attributed to the reordering of membrane phospholipids and reduced membrane permeability [152]. Kumar et al. also investigated the passage of RONS through non-oxidized and hydroperoxidized POPC membranes in the presence of cholesterol [98]. The overall effect of membrane oxidation and cholesterol on RONS permeation was consistent with previous studies, suggesting that hydrophobic species can pass more easily, particularly when the membrane is oxidized [75,81,152,172]. Comparing the free energy profiles obtained for ROS permeation across DOPC [81] and cholesterol-rich POPC [98], as well as their hydroperoxidized forms, no significant difference in the energy barriers for ROS was observed. This indicates that POPC could be more permeable to ROS if cholesterol was not incorporated.

**Figure 7:**
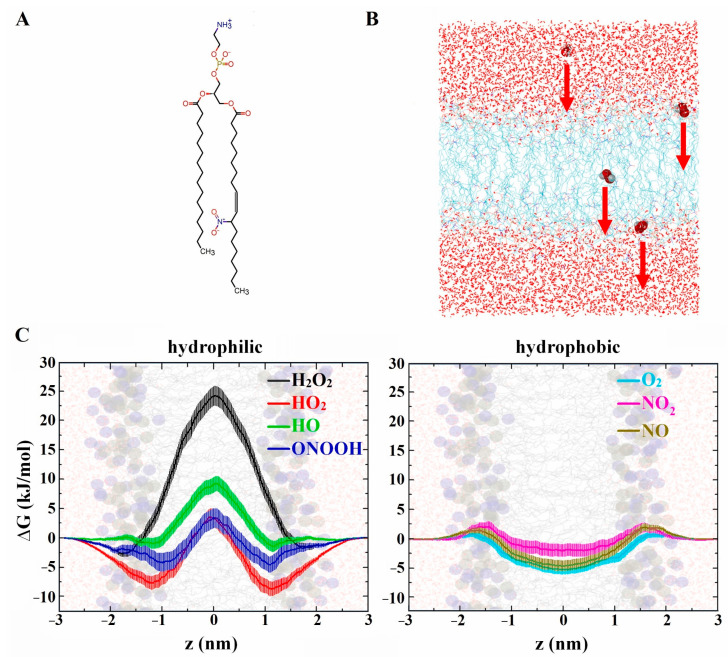
(**A**) Schematic representation of the chemical structure of nitrated POPC lipid. (**B**) The position of H_2_O_2_ as an example of RONS located within the membrane. The red arrows indicate the direction of pulling of H_2_O_2_ into the membrane. (**C**) Symmetrized free energy profiles for the passage of hydrophilic RONS (**left**) and hydrophobic RONS (**right**) across the membrane. Reprinted/adapted with permission from Ref. [48]. 2021, Elsevier.

In addition to membrane oxidation, membrane nitration can also occur through the action of CAP-generated RNS. Oliveira et al. [48] investigated the permeation of hydrophilic and hydrophobic RONS through nitrated POPC [71] and compared their results with other studies on the passage of RONS through non-oxidized [184] and oxidized POPC [75,81]. The permeation free energy barrier for hydrophilic species (H_2_O_2_, HOO^•^, ^•^OH, ONOOH) and hydrophobic species (O_2_, ^•^NO_2_, ^•^NO) through 100% nitrated POPC, obtained through US simulations, showed that the nitrated membrane exhibited higher permeability for hydrophobic RONS compared to hydrophilic species (see Figure 7) [48].

When compared with other studies on the hydroperoxidation of 50% POPC, the nitrated membrane demonstrated similar permeability for hydrophilic species but greater permeability than non-oxidized and hydroperoxidized POPC for hydrophobic species. In a recent MD study, Abduvokhidov et al. investigated the transport properties of RONS across membranes with varying degrees of lipid nitro-oxidation [185]. The results, obtained using US simulations, revealed that certain RONS, namely ^•^NO, ^•^NO_2_, N_2_O_4_, and O_3_, exhibited higher penetration capabilities across both native and nitro-oxidized membranes compared to HNO_3_, *s-cis*-HONO, *s-trans*-HONO, H_2_O_2_, HOO^•^, and ^•^OH. They also found that nitro-oxidation of the membrane had an insignificant impact on the free energy barriers for the former RONS, whereas it lowered these barriers for the latter RONS, thereby enhancing their permeation through nitro-oxidized membranes. In general, all CAP-generated RONS, either directly or indirectly, can penetrate cell membranes and induce intracellular changes through the nitro-oxidation of cell organelles and proteins, which can be effective in bio-plasma therapy.

## 3. Non-Reactive MD Simulations of Proteins

**Figure 8:**
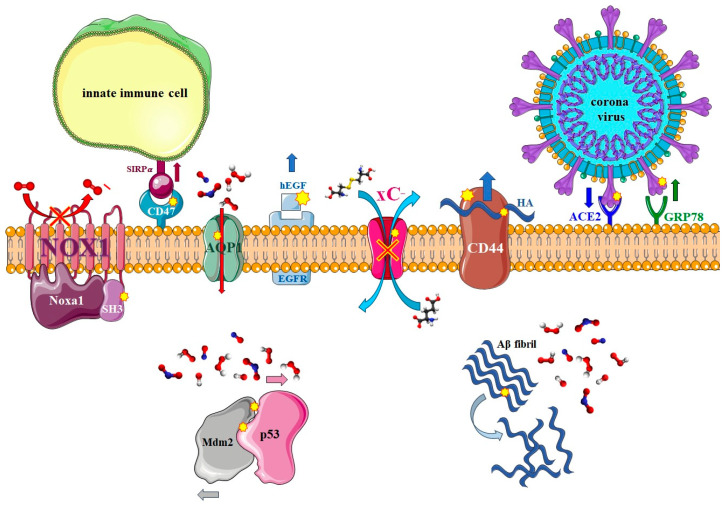
Schematic representation of several important proteins modified by (CAP-generated) RONS. Detailed discussions of these modifications are presented in the following sections. Briefly, from left to right: modification (namely, oxidation) of membrane proteins can lead to the denaturation of Noxa1 SH3 and subsequent inactivation of NOX1, resulting in the inhibition of cancer cell proliferation [198]; reduction in the binding affinity of CD47 to SIRPα, thereby decreasing the chance of cancer cells evading immune cells [192]; induction of slight changes in RONS translocation across AQP1, with a lesser impact on their permeability into cells [80]; disruption of the interaction between hEGF and EGFR, potentially interfering with cellular signaling pathways associated with cancer cell proliferation [36]; interruption of cystine uptake by xC^−^ antiporter, ultimately triggering cancer cell apoptosis [190]; reduction in the binding free energy between HA and CD44, inhibiting the signaling pathways related to cancer cell proliferation [199]; and strengthening (weakening) the interaction between the coronavirus spike protein and ACE2 (GRP78), crucial for the treatment of viral infections caused by SARS-CoV-2 [204]. CAP can also reduce the binding between Mdm2 and p53, an essential factor for tumor suppression [202], and disaggregate Aβ fibrils, contributing to the treatment of Alzheimer’s disease [195].

Proteins are the most crucial components of living systems and are essential for the growth and maintenance of organisms. If proteins lose their functionality, all cellular signaling pathways are disrupted [108]. Proteins are generally categorized into nine functional classes: antibodies, enzymes, hormones, motor proteins, receptors, transport proteins, structural proteins, signaling proteins, and storage proteins [186]. The functions of proteins are derived from their structures, which include the amino acid sequence and the overall three-dimensional shape. Depending on their specific functions, proteins possess an active site domain that contains specific amino acid residues crucial for their activities [187]. Modifications in protein structures, such as amino acid mutations [188,189] or oxidation and nitration induced by RONS interactions [36,80,190,191,192,193,194,195,196], can directly or indirectly affect the active sites and, subsequently, the protein functions [197]. Understanding the impact of such modifications on protein structure and function at the atomic scale is challenging to investigate experimentally. MD simulations provide a valuable tool to study these effects by bridging the gap between structure and dynamics, particularly for non-bonded interactions. This review focuses on papers that have employed MD simulations to investigate the effects of oxidation and nitration of amino acid residues in proteins due to interactions with CAP on their functions. Many of these proteins are significant in cancer therapy, as CAP can selectively kill certain types of cancer cells while causing less damage to normal cells, which makes this method appropriate for cancer therapy [36,80,190,191,192,194,198,199,200,201,202]. This selectivity may be attributed to specific proteins and phospholipids (mentioned earlier) found in cancer cells, which are adapted to the high metabolic activity of these cells. In many cases, the presence of mutated, overexpressed, or underexpressed proteins compared to their normal counterparts plays a key role in the selectivity of CAP treatment. Additionally, some of these proteins, along with others relevant to Alzheimer’s disease [195], wound healing [36,194], and viral and bacterial infections [203,204,205], are discussed here. The consistency between MD simulations and experimental results in most studies demonstrates that MD simulations are a reliable and cost-effective method for investigating and predicting the effects of CAP on protein structures, and subsequently on cells and tissues. This section specifically explores the impact of nitro-oxidation on two categories of proteins: membrane proteins and intracellular proteins, elucidating their structure and function at the atomic scale and highlighting the significance of CAP as an extracellular RONS source. Figure 8 schematically shows some of the important proteins that are modified by (CAP-generated) RONS and whose modifications are useful in bio-plasma therapy. These proteins are discussed in detail in the following sections.

### 3.1. Nitro-Oxidation of Protein Amino Acids

During CAP treatment, amino acid residues of proteins can undergo nitro-oxidation through reactions with RONS. Since non-reactive MD simulations cannot capture bond breakage and formation, computational studies using non-reactive MD simulations should consider the oxidation and nitration products of amino acid residues obtained from experimental studies. Takai et al. [206] conducted a study on the chemical modification of amino acids in solution using a low-frequency plasma jet. They identified different oxidized products depending on the duration of CAP exposure. The modified products of amino acids and their abundance were found to be dependent on the plasma devices and the environmental condition. For instance, Zhou et al. used a micro-plasma array device and observed the modification of amino acids for 30 min, resulting in a greater variety of oxidized and nitrated products [207]. Furthermore, Wenske et al. recently investigated the changes in amino acid residues of different types of peptides using two different devices, i.e., an argon-driven MHz-jet kINPen and the helium-driven RF-jet COST-Jet [208,209]. Although the reactivity of amino acids to nitro-oxidation was consistent with previous studies, the abundance of products was different not only from previous studies but also from each other. Therefore, to specify the modified amino acids in MD simulations, the product produced by a specific device should be considered, which helps to ensure that MD results can be qualitatively consistent with experimental data.

**Figure 9:**
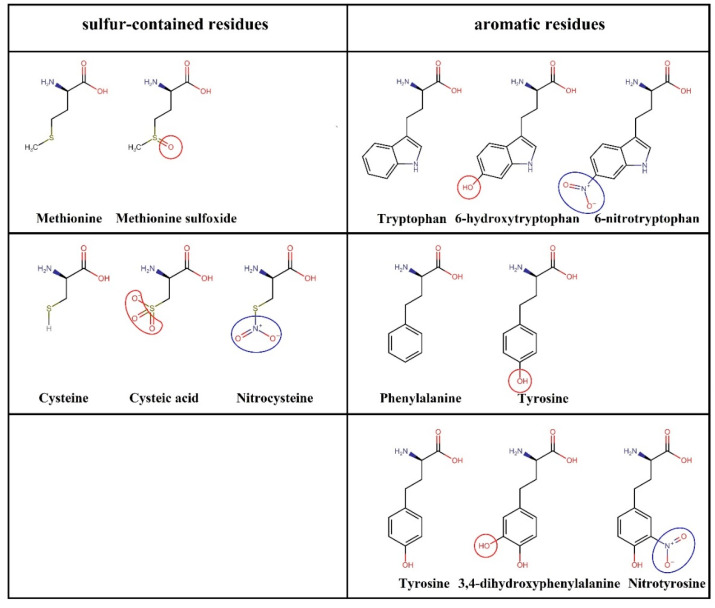
Chemical structure of non-oxidized and nitro-oxidized forms of sulfur-containing (**left**) and aromatic (**right**) residues. Oxidized and nitrated groups are shown in red and blue circles, respectively.

The reactivity of amino acid residues towards oxidation varies, and higher doses of CAP and more reactive RONS are required to nitro-oxidize certain amino acid residues. Among the 20 amino acids, methionine (Met) and cysteine (Cys), which contain sulfur, are the most reactive and readily undergo oxidation to form methionine sulfoxide and cysteic acid, respectively (Figure 9, left column) [206,207]. Aromatic residues such as tryptophan (Trp), phenylalanine (Phe), and tyrosine (Tyr) can mostly be oxidized to 6-hydroxytryptophan, tyrosine, and 3,4-dihydroxyphenylalanine, respectively [206,207] (Figure 9, right column). Other amino acid residues require very high doses of CAP for oxidation, but some of their oxidation products considered in protein oxidation studies by CAP-generated RONS include 2-oxo-histidine, pyroglutamic acid, 4-hydroxyglutamine, allysine, 3-hydroxyvaline, 4-hydroxyleucine, and 3-hydroxyasparagine, derived from histidine (His), proline (Pro), glutamine (Gln), lysine (Lys), valine (Val), leucine (Leu), and asparagine (Asn), respectively [36,206]. In addition to oxidation, CAP can also nitrate certain amino acid residues. Protein nitration is generally rare, but Cys, Trp, and Tyr can be nitrated to form nitrocysteine, 6-nitrotryptophan, and nitrotyrosine, respectively [210] (see Figure 9). Therefore, when investigating the effect of protein nitro-oxidation on their function, the aforementioned modified amino acids are used in MD simulations.

### 3.2. Effect of Nitro-Oxidation on the Properties of Membrane Proteins

Membrane proteins are located within lipid rafts in the cell membrane [211]. During exposure to CAP, they are among the first group of proteins that can undergo nitro-oxidation by RONS generated by CAP. Based on their function, membrane proteins are classified into three main categories: transmembrane proteins, membrane enzymes, and membrane receptors. In this section, we will discuss the nitro-oxidation of selected membrane proteins that play a role in bio-plasma therapy.

#### 3.2.1. Transmembrane Proteins

Transmembrane proteins are integral components of the cell membrane. They play crucial roles in various biological processes, including cell signaling, transport of molecules across the membrane, and cell adhesion. Transmembrane proteins can be classified into various groups based on their structure, topology, location, and function [108]. Since these proteins are located on the cell membrane, they are among the first to be affected by CAP. In this section, we will review the nitro-oxidation of five transmembrane proteins—AQP1 [80], xC^−^ antiporter [189,190], CD44 [199], CD47 [192], and EGFR [36,194]—that have undergone structural changes or experienced alterations in their related ligands due to RONS generated by CAP. These proteins play a significant role in CAP treatment and have been investigated using MD simulations.

One important group of transmembrane proteins involved in CAP therapy is aquaporins (AQPs), which facilitate the transport of water molecules and other solutes across the cell membrane. AQPs also play a crucial role in CAP treatment as they can uptake RONS generated by CAP into the cell. Excessive RONS absorbed by AQPs can induce nitro-oxidative stress within the cell, leading to damage to intracellular biomolecules and signaling pathways, ultimately resulting in cell death [212]. AQPs are often overexpressed in various types of cancer cells, including breast [213,214], lung [215], ovarian [216,217], and colorectal cancer [218,219]. This overexpression allows AQPs to increase the intracellular RONS level in cancer cells compared to normal cells, making CAP more detrimental to cancer cells than to normal cells [220,221]. Furthermore, RONS generated by CAP can oxidize both AQPs and the phospholipids in the surrounding membrane, potentially interfering with RONS transport mediated by AQPs [212,222,223]. Fortunately, MD simulations conducted by Yusupov et al. [80] on the transport of RONS through AQP1 indicated that the oxidation of AQP1 or its surrounding lipid membrane had no significant effect on transport. Free energy profiles obtained from US simulations demonstrated that larger molecules such as H_2_O_2_ and ^•^NO_2_ were more challenging to transport through AQP1 compared to ^•^NO and ^•^OH under all conditions. In general, the authors suggested that hydrophobic RONS such as O_2_, ^•^NO, and ^•^NO_2_ most likely pass through the lipid membrane, where lipid oxidation can occur, due to their higher barrier through AQPs (i.e., ~5–12 kJ/mol vs. ~1 kJ/mol for the lipid membrane) [81]. On the other hand, hydrophilic RONS like ^•^OH and particularly H_2_O_2_ permeate the cell through AQPs, as they encounter a significantly higher barrier when crossing the lipid membrane (~15–30 kJ/mol) [75,81,184,224], particularly in the non-oxidized (intact) lipid membrane. Thus, the presence of AQP1 can improve the transmembrane efficiency of hydrophilic ROS [225]. This is consistent with the conclusion of Cui et al. on the differences in the transmembrane transport of hydrophilic ROS and hydrophobic RNS [226]. These findings indicate that both the lipid membrane and AQPs are permeable to (CAP-generated) RONS, particularly in cancer cells, resulting in an increase in intracellular RONS levels after CAP treatment, leading to elevated nitro-oxidative damage to intracellular organelles and proteins.

**Figure 10:**
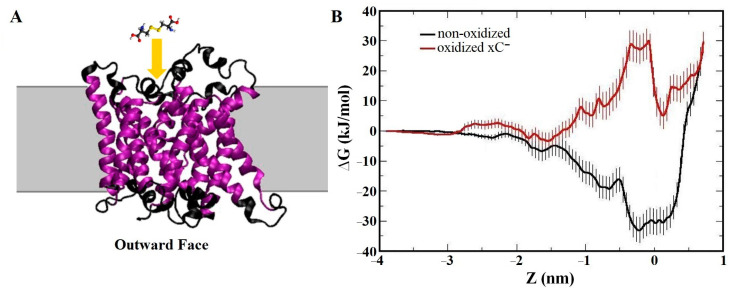
(**A**) Schematic illustration of the cystine translocation from xC^−^ antiporter. The yellow arrow indicates the direction of separation of cystine toward outward face of xC^−^ antiporter protein. (**B**) Free energy profiles for the translocation of cystine across the non-oxidized (black curve) and oxidized (red curve) xC^−^. Reprinted/adapted with permission from Refs. [189,190]. 2019, Elsevier.

Another vital transmembrane protein that is overexpressed in many cancer cell types and is essential for cell viability is the xC^−^ antiporter [227]. This antiporter is responsible for transporting cystine (the oxidized dimeric form of Cys) from the extracellular environment into the cell while exporting glutamate from the cell to the extracellular space [228,229]. Cystine is rapidly converted to Cys inside the cell and contributes to the biosynthesis of glutathione, which plays a crucial role in combating intracellular oxidative stress [228]. Certain cancer cells, such as lymphoma and leukemia cells, lose their ability to synthesize Cys internally, making cystine uptake by the xC^−^ antiporter crucial for their survival. Consequently, these cancer cells exhibit overexpression of this antiporter on their membrane [230]. Inhibition of the xC^−^ antiporter by RONS generated by CAP can result in intracellular Cys starvation, particularly in cancer cells, affecting glutathione levels. This, in turn, can inhibit cell growth and ultimately induce apoptosis [228]. Experimental studies on the xC^−^ antiporter have shown that the mutation of Cys_327_, located near the protein channel and the extracellular environment, to alanine (Ala) can disrupt normal cystine transport, indicating the significance of Cys_327_ in this process [231]. Cys_327_ is highly reactive and easily oxidized to cysteic acid by RONS [206]. MD simulations conducted by Ghasemitarei et al. [190] demonstrated that the oxidation of Cys_327_ can impact the channel structure and hinder cystine uptake. Free energy profiles obtained from US simulations of cystine translocation across the outward face of the xC^−^ antiporter revealed that Cys_327_ oxidation created a barrier of 33.9 kJ/mol, resulting in reduced cystine permeation rates (see Figure 10). Analysis of the protein channel size indicated that the channel was nearly closed in the vicinity of oxidized Cys_327_, making it more difficult for cystine to be taken up. Thus, the inhibition of the xC^−^ antiporter by (CAP-generated) RONS results in the depletion of cystine within cancer cells, leading to a reduction in intracellular glutathione levels. The decrease in glutathione levels triggers an elevation in intracellular oxidative stress, ultimately inhibiting cell growth and inducing apoptosis specifically in cancer cells [190].

**Figure 11:**
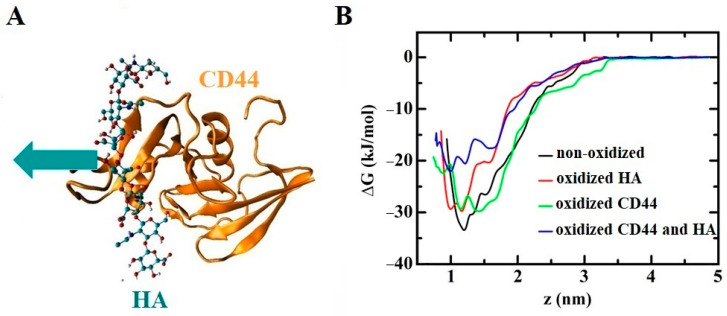
(**A**) Schematic illustration of the CD44–HA complex. The cyan arrow indicates the direction of separation of HA sugar chain from CD44 protein. (**B**) Free energy profiles of dissociation of HA from CD44, where they are both in non-oxidized form (black curve), only HA is oxidized (red curve), only CD44 is oxidized (green curve), and both are oxidized (blue curve). Reprinted/adapted with permission from Ref. [199]. 2012, Elsevier.

Cluster of differentiation 44 (CD44) is a transmembrane protein that is known to be overexpressed in various types of cancer cells [232]. Its increased expression is often associated with cancer progression, metastasis, and poor prognosis. CD44 plays important roles in cell adhesion, migration, and signaling, and its overexpression is believed to contribute to cancer cell invasion and metastasis. It facilitates cell-to-cell and cell-to-matrix attachment of cancer cells, promoting cell proliferation, differentiation, invasion, and migration [233]. The main ligand of CD44 is hyaluronan (HA), located in the tumor extracellular matrix, which activates signaling pathways associated with increased cell metastasis [234]. Yusupov et al. conducted an experimental study demonstrating that reducing the interaction between HA and CD44 through CAP treatment could disrupt signaling pathways driving tumor progression [199]. They also investigated the oxidative damage of HA and CD44 by CAP-generated RONS and its effect on their binding affinity using both reactive (i.e., DFTB) and non-reactive MD simulations. The DFTB-MD was used to understand the chemical reaction mechanism of HA oxidation by RONS, and the resulting oxidized HA was used in the non-reactive MD simulations. The dissociation free energy profiles obtained by US simulations (Figure 11) indicated that oxidation of both CD44 and HA weakened their interaction, thereby inhibiting the signaling pathways of cancer cell proliferation. Structural analysis revealed that oxidation of CD44 destabilized its structure due to the breaking of disulfide bonds between Cys_77_-Cys_97_ and Cys_28_-Cys_129_, leading to decreased binding to HA. Consequently, the dissociation of CD44 and HA caused by CAP-generated RONS could be a crucial mechanism for cancer treatment based on oxidative stress [199].

In addition to apoptotic cell death mediated by (CAP-generated) RONS, CAP can also induce immunogenic cell death by enhancing the ability of the immune system to detect and eliminate cancer cells [18]. In cancer immunotherapy using CAP, immunosuppressive surface proteins and checkpoints that are often overexpressed on the membrane of cancer cells can be targeted by CAP [235]. One such checkpoint, CD47, is a transmembrane protein overexpressed on the membrane of cancer cells that binds to signal-regulatory protein alpha (SIRPα) of innate immune cells [236,237,238], sending a “don’t eat me” signal and helping the escape of cancer cells from the immune system [239]. Lin et al. conducted a combined experimental and computational study to investigate the effect of CAP on CD47 [192]. Their simulation results showed that oxidation of CD47 reduced its binding affinity to SIRPα on innate immune cells, thereby decreasing the chance of cancer cells evading immune cells [192]. The free energy profiles obtained by US simulations showed that the free energy of CD47-SIRPα separation reduced by ~45 kJ/mol after oxidation, and due to the conformational changes of oxidized CD47, the Lys_39_-Asp_100_ and Lys_41_-Asp_100_ salt bridges between CD47 and SIRPα were broken. The authors concluded that the dissociation of oxidized CD47-SIRPα helps enhance tumor immunogenicity and support anti-cancer immune response, supporting its role in CAP-mediated cancer immunotherapy [192].

Epidermal growth factor receptor (EGFR) is another transmembrane glycoprotein that plays an important role in cancer treatment [240] and wound healing [241], and it has numerous ligands for its activation [242,243]. This protein plays a crucial role in cell proliferation, survival, and differentiation, which is useful for wound healing and is part of the healing process. However, in cancer cells, this mechanism acts against the treatment and allows the cancer cells to progress in the body [36,194,244,245,246]. Overexpression of EGFR has been observed in many cancer types, which affects the cell cycle by inducing these mechanisms [244,247]. Human epidermal growth factor (hEGF), a small ligand, is one of its activating ligands [248]. It appears that this ligand indirectly possesses cancer-inducing abilities by stimulating cytoprotection, chemotaxis, mutagenesis, and mitogenesis, leading to the promotion of cancer growth [249]. However, these stimuli are effective in wound healing. Therefore, any changes to hEGF caused by (CAP-generated) RONS could be useful for one of two treatments: wound healing or cancer treatment [36]. In this case, the timing of CAP treatment and the type of RONS become important factors that can affect the level and type of hEGF modifications [61]. If too many residues are nitro-oxidized by RONS, it can completely disrupt the normal function of hEGF [36,194], which can be useful in cancer therapy. Conversely, small changes in the protein structure can maintain its normal function, which is beneficial in the wound-healing process [36]. The modification of hEGF by RONS affects the stability and flexibility of its structure and subsequently its binding to EGFR [36]. The effects of different levels of hEGF oxidation on its binding to EGFR and its structural changes have been studied by Yusupov et al. [36] using molecular mechanics poisson-boltzmann surface area (MMPBSA) and structural analysis (i.e., root-mean-square deviation (RMSD), solvent accessible surface area (SASA), and principal component analysis (PCA)). They found that higher levels of oxidation significantly reduced the binding affinity between the two proteins (EGFR and hEGF) and made hEGF more unstable with higher fluctuations. Denaturation of hEGF as a result of increased levels of oxidation is associated with the breaking of disulfide bonds at higher levels [36]. The nitrosylation of hEGF was also studied by Razzokov et al. [194]. Again, different levels of hEGF nitrosylation were considered, and the stability and fluctuation of the structures were compared. The results revealed that the nitrosylation of hEGF does not disrupt its structure, even if all three disulfide bonds are broken at high nitrosylation levels. All systems remained stable as the nitrosylation of amino acids made them more hydrophobic, causing them to become more compact compared to the native structure as they do not interact favorably with water. Therefore, it can be concluded that nitrosylation of hEGF does not cause any fundamental changes in its structure and, subsequently, its function, which can be useful for wound healing [194]. In contrast, the oxidation of hEGF had significant effects on its structure, binding to its receptor, and function, as oxidation made hEGF more hydrophilic, allowing it to become more accessible to water and causing further changes in the hEGF structure [36]. Based on the simulation results, lower oxidation of hEGF and its lesser effect on binding affinity to its receptor do not disrupt the normal signaling pathways in the cell and the mechanism of cell proliferation, which explains why CAP can be useful in wound healing if the CAP treatment time is short [37]. On the other hand, higher oxidation of hEGF and the loss of its ability to bind to its receptor may disrupt the cell signaling pathways related to the cell proliferation mechanism. This could provide a good explanation as to why longer CAP treatment times can be beneficial in cancer treatment.

#### 3.2.2. Enzymatic Proteins

In this section, we focus on three proteins that are overexpressed in cancer cells and play a role in their proliferation, invasion, and growth. These proteins, NOX [198], PLA2 [250], and FAK [201], have been identified as potential targets for cancer therapy, particularly using CAP. Recent studies have shown that their oxidation by CAP can disrupt their normal function and can have a therapeutic effect on cancer treatment.

NADPH oxidase (NOX) enzymes are involved in various physiological functions in cells, including cell proliferation, differentiation, growth, and tissue regeneration [251,252,253]. Among NOX enzymes, NOX1 produces superoxide (O_2_^•−^) through the interaction between Nox organizer 1 (Noxo1) and Nox activator 1 (Noxa1 SH3) [254]. ROS produced by NOX1 play a significant role in cancer development and metastasis [255,256]. Attri et al. studied the effect of oxidation on the function of Noxa1 SH3, a subunit of NOX1, by CAP-generated RONS [198]. The MD simulations of the oxidized form of Noxa1 SH3 included four oxidized amino acids: Gln_413_, Cys_430_, Cys_441_, and Val_432_. Structural analysis of the oxidized structure revealed that the oxidation of Noxa1 SH3 made it unstable with higher flexibility, which aligned with experimental investigations showing that CAP treatment caused denaturation of Noxa1 SH3 and subsequent inactivation of NOX1, leading to the inhibition of cancer cell proliferation, differentiation, growth, and tissue regeneration [198].

Phospholipase A2 (PLA2) is an enzymatic membrane protein responsible for hydrolyzing phospholipids and damaging lipid bilayers, thereby increasing membrane permeability [257,258,259]. PLA2 releases lipid mediators such as lysophosphatidic acid, which acts as a signaling molecule and promotes cellular processes including proliferation, survival, invasion, and growth, especially in cancer cells [260]. Overexpression of PLA2 has been observed in various types of cancer cells [261,262]. PLA2 also plays an important role in diseases such as asthma [263,264], multiple sclerosis [265], schizophrenia [266,267,268], autism [269], and bipolar disorders [270,271]. Therefore, regulating the activity of PLA2 may be effective in controlling these diseases. PLA2 binds to the membrane through its interface site and activates its catalytic site [257,272]. Nasri et al. conducted a combined experimental and computational study on the CAP treatment of PLA2 [250]. They found that the changes in membrane permeability were negligible after CAP-treated enzymes, indicating that oxidized PLA2 was unable to hydrolyze phospholipids. Structural analysis of the oxidized PLA2 revealed that highly reactive amino acids, such as Cys and Met, were not modified by CAP, possibly due to their limited exposure to RONS. However, Trp_128_, located on the outer surface of PLA2 and involved in binding to different ligands, was extensively oxidized, resulting in the opening of its indole ring. This oxidation reduced its ability to bind to ligands, particularly membranes. Trp_128_ played a crucial role in the separation, which was investigated at the atomic scale using MD simulations and molecular docking. Although the oxidation of Trp_128_ had no effect on the overall structure and size of PLA2, especially in its active site, molecular docking results indicated that Trp oxidation decreased the hydrogen bonds and interaction energy between PLA2 and PE. Additionally, the π-interaction between the indole ring of Trp_128_ and PE was disrupted after oxidation [250].

Focal adhesion kinase (FAK), a non-receptor tyrosine kinase, is the last enzymatic protein mentioned in this section. It plays a crucial role in cell-cell and cell-matrix interactions, thereby controlling important cellular processes, such as cell adhesion [273], migration [274], and proliferation [275]. The enzymatic activity of FAK is regulated by its catalytic and FERM (Four-point-one, ezrin, radixin, moesin) domains [276,277]. The strong interaction between the catalytic and FERM domains keeps FAK in an autoinhibited state, protecting its activation loop from phosphorylation by Src kinase [278]. Disruption of the FERM-catalytic domain interaction, whether due to intracellular signaling or external factors such as CAP exposure, leads to the activation of FAK and increased cell migration and proliferation [276,277,279]. Han et al. demonstrated that low doses of CAP can activate FAK, promoting wound healing, while high doses of CAP fail to activate FAK, which may be relevant in cancer therapy [201]. The activation of FAK may be associated with the disruption of the oxidized catalytic-FERM interaction. Thus, Han et al. investigated the structural changes of the oxidized catalytic domain using MD simulations at the atomic scale, which was not feasible experimentally. Oxidation of highly reactive amino acids, such as Cys and Met, equivalent to low-dose CAP oxidation of the catalytic domain, destabilized the catalytic domain and increased its fluctuation. This disruption of the FERM-catalytic domain interaction resulted in the phosphorylation of Tyr_397_ and subsequent enhancement of the enzymatic activity of FAK, which is beneficial for promoting the wound healing process [201]. The inactivation of FAK by high doses of CAP could be attributed to the oxidation of other residues in the catalytic domain, leading to increased binding between the FERM and catalytic domains. However, this aspect has not been studied at the atomic level and requires further investigation.

Overall, the MD simulations used to study the effect of CAP oxidation on Noxa1 SH3, PLA2, and FAK provided valuable atomic-scale insights into how oxidation disrupts their normal functions. These findings align with the reduction of cancer cell proliferation and growth, making them potentially valuable in cancer treatment.

#### 3.2.3. Membrane Receptors

Membrane receptors are a type of proteins that are either attached to or embedded in cell membranes and are activated through interaction with ligands. Among the various receptors with different functions, angiotensin converting enzyme 2 (ACE2) is a crucial receptor mentioned in this section, playing a significant role in the viral infection caused by SARS-CoV-2 [280]. ACE2 is a zinc metalloenzyme attached to the cell membranes of various organs such as the heart, lungs, arteries, kidneys, and intestines, and it has enzymatic functions within cells [281]. This ectoenzyme (i.e., an enzyme located on the outer surface of the cell membrane) acts as a gateway for SARS-CoV-2, as the S-glycoprotein of the virus attaches to this receptor [280,282]. The S-glycoprotein, located on the outer surface of the viral particle, can bind to cells through its receptor binding domains (RBDs), specifically the RBD of the S1 subunit [282]. Discovering methods to decrease or inhibit this attachment can be useful in reducing viral infections. CAP has demonstrated antiviral properties [283] and effectively deactivates SARS-CoV-2 on various surfaces [284]. Guo et al. experimentally studied the interaction between CAP-generated RONS and the RBD domain, as well as its attachment to ACE2 [285]. They showed that CAP treatment caused fragmentation of the RBD domain, separating it from ACE2. Although non-reactive MD simulations cannot study RBD fragmentation, Attri et al. investigated the effect of high doses of RBD oxidation on its binding to ACE2 using MD simulation [203]. They selectively oxidized amino acids in the RBD that play a crucial role in the interaction with ACE2. Two oxidized systems were designed: in the first system, they oxidized amino acids directly interacting with ACE2, and in the second system, they oxidized additional amino acids in the vicinity of ACE2. Structural analysis of the oxidized RBD-ACE2 complex revealed that RBD oxidation made the complex unstable with higher fluctuations. However, the analysis of the binding affinity of the RBD-ACE2 complex using MMPBSA showed that although the oxidized RBD and ACE2 had slightly dissociated in the first system, they exhibited much stronger binding affinity in the second system compared to the natural state. Therefore, their MD simulation results indicated that RBD oxidation alone, without considering RBD nitration, would not be helpful in reducing its binding to ACE2 and consequently inactivating the virus [203]. Nitration of RBD, particularly by ONOO^−^, leads to RBD fragmentation [204,285], which could be useful in inhibiting the binding of SARS-CoV-2 to cells [204]. However, RBD fragmentation cannot be studied using non-reactive MD simulations. Although not in the context of CAP treatment, Ghasemitarei et al. also examined the effect of Cys oxidation of RBD on its attachment to ACE2 [204], and their results confirmed the previous claim. Through MD simulations, they investigated the oxidation of Cys, a highly reactive amino acid targeted by RONS, in the RBD. Among the four disulfide bonds between Cys residues in the RBD domain, two pairs, specifically Cys_379_-Cys_432_ and Cys_480_-Cys_488_, were broken and oxidized to cysteic acid. The non-bonded energy between RBD and ACE2 indicated that RBD oxidation increased the attractive interaction between RBD and ACE2. Additionally, the difference in the binding free energy of RBD and ACE2 before and after oxidation, obtained through slow-growth free energy simulations, was equal to −55.1 ± 31.5 kJ/mol, confirming an increased binding affinity after RBD oxidation. In summary, their results also explained that oxidation of RBD alone, without considering nitration, would not be beneficial in preventing RBD binding to ACE2 and subsequently inhibiting viral infection. Therefore, further studies on the effect of RBD nitration are necessary to explore the impact of CAP-generated RONS on the binding affinity of the RBD-ACE2 complex [204]. Another membrane receptor involved in SARS-CoV-2 infection is glucose-regulating protein 78 (GRP78, also known as binding immunoglobulin protein) [286]. GRP78 is a master chaperone protein normally located in the lumen of the endoplasmic reticulum [287]. Under cellular stress, it can be overexpressed, and excessive GRP78 can be translocated to the cell membrane [286]. Experimental studies have shown that inhibiting membrane GRP78 with certain ligands reduces SARS-CoV-2 viral infection [288,289,290]. Furthermore, using MD simulations, it was demonstrated that Cys oxidation of RBD could separate RBD from GRP78 [204]. The oxidized RBD was the same as the oxidized RBD in the RBD-ACE2 complex (mentioned above). Although GRP78 is not the primary receptor for RBD, it plays a crucial role in anchoring the virus to the cell membrane and facilitating RBD binding to ACE2. Therefore, dissociation of RBD from GRP78 may reduce the probability of RBD-ACE2 binding and subsequently reduce viral infectivity [204].

### 3.3. Effect of Nitro-Oxidation on the Properties of Intracellular Proteins

As mentioned in the previous section, CAP-generated RONS can penetrate cell membranes and increase intracellular levels of RONS, leading to nitro-oxidative damage to intracellular proteins. This section focuses on the modification of certain proteins that play key roles in diseases such as cancer [191,200,202,291], chronic wounds [200,202], and Alzheimer’s disease [195] by CAP-generated RONS, with the aim of treatment.

In CAP therapy, particularly in cancer treatment, studying the effects of oxidation or nitration of proteins involved in regulating cell proliferation, differentiation, division, and growth is of great interest. This research can provide important insights into the impact of CAP treatment on controlling the spread of cancer cells in the body. The effects of CAP on five types of these proteins, namely hEGF, CD44, NOX1, PLA2, and FAK, have already been discussed (see above). Another protein that plays a crucial role in controlling cell division is p53 [292]. This protein, found in the nucleus of cells, acts as a tumor suppressor by regulating the cell cycle and inhibiting malignant transformations [293,294,295]. Murine double minute 2 (Mdm2), found in various types of cancers such as liver [296], breast [297,298], lung [299], and colorectal cancer [300,301,302], can inactivate p53 by binding to its hydrophobic α-helix, which contains 70% of the atoms at the nonpolar interface [303,304]. Finding methods or agents to inhibit the formation of the p53-Mdm2 complex could be useful in enhancing the anticancer activity mediated by p53. Attri et al. discovered that CAP could be an effective method for inhibiting the formation of the p53-Mdm2 complex [202]. They studied the binding affinity of the p53-Mdm2 complex before and after possible oxidation by CAP-generated RONS using MD simulations. In their study, they investigated the oxidation of both p53 and Mdm2 on the binding affinity of the p53-Mdm2 complex. Structural analysis revealed that the flexibility of all oxidized systems, particularly when only Mdm2 was oxidized, increased compared to the non-oxidized system. Their results demonstrated that oxidized Mdm2 was unable to inhibit non-oxidized p53 [202]. However, more research on the binding free energy of the two proteins is needed to confirm that the oxidation of the p53-Mdm2 complex can reduce their binding affinity and ultimately lead to the disinhibition of p53 by oxidized Mdm2.

In addition to proteins that play key roles in cell division, proliferation, and growth, proteins that protect cells from oxidative stress, such as redox-sensitive proteins, are also important in cancer therapy. These proteins function as regulators of the cellular redox balance, mainly through oxidative modification of redox-sensitive residues, including Cys residues [305,306,307,308]. Cytoglobin (CYGB) and glutathione peroxidase 4 (GPX4) are two members of this protein family whose modification by CAP-generated RONS can be effective in cancer therapy [291,309,310], and they have been studied at the atomic scale using MD simulations [191]. Experiments have shown that CYGB can scavenge intracellular RONS and protect cells against oxidative and nitrative stress [311,312,313]. CYGB, a member of the globin family that contains a heme group (which includes iron), plays an important role as a tumor suppressor due to its differential concentration in cancer cells and subsequent scavenging of RONS [314,315,316]. Generally, the intracellular level of RONS is higher in cancer cells compared to normal cells, which is associated with tumor proliferation, angiogenesis, and metastasis [316]. Therefore, increasing the enzymatic activity of CYGB as a result of CAP-generated RONS can be beneficial in cancer treatment, as demonstrated by De Backer et al. [191]. Through MD simulation and molecular docking, they discovered that the formation of a disulfide bond between a pair of Cys residues (Cys_38_ and Cys_83_) by CAP-generated RONS increased the accessibility of the binding pocket to the heme group of CYGB, which influenced its ligand-binding properties. These conformational changes in CYGB resulted in different interactions with ligands, such as suppressor drug-like molecules, which can affect intracellular oxidative stress [191]. Additionally, Razzokov et al. demonstrated that the mutation of Lys_80_ to Ala (L80A) also increased the accessibility of the CYGB binding pocket to the heme group, even in the absence of disulfide bond formation [200]. However, disulfide bond formation further enhanced the open accessibility of the binding pocket to the heme group. In contrast, their previous study [191] showed that without this mutation and disulfide bond formation, the CYGB binding pocket was not accessible to the heme group [200]. In general, the combination of CYGB mutation and disulfide bond formation between Cys_38_ and Cys_83_ through CAP-generated RONS increased the enzymatic activity of CYGB as a cytoprotective protein, which could be beneficial in cancer therapy.

Another redox-sensitive protein, GPX4, acts as a phospholipid-hydroperoxide and protects cells against membrane lipid peroxidation [317]. When the enzymatic activity of GPX4 is inhibited and (CAP-generated) RONS interact with the cell membrane, it increases the level of Fe^2+^ and lipid peroxidation, ultimately inducing ferroptotic cancer cell death [318,319]. Ferroptosis is an iron-catalyzed necrosis characterized by the accumulation of lipid peroxides [320]. Cancer cells have a higher iron requirement for growth compared to normal cells, making them more susceptible to ferroptosis [319,321]. Kumar et al. demonstrated that the oxidation of GPX4 by CAP-generated RONS could inhibit its enzymatic activity, disrupting the lipid repair system [291]. This, in turn, leads to ferroptotic cell death in cancer cells during CAP exposure. Through MD simulations, they studied the structural changes in the catalytic active pocket of oxidized GPX4 and found that oxidation increased the flexibility of the pocket, resulting in decreased stability and loss of enzymatic activity [291].

**Figure 12:**
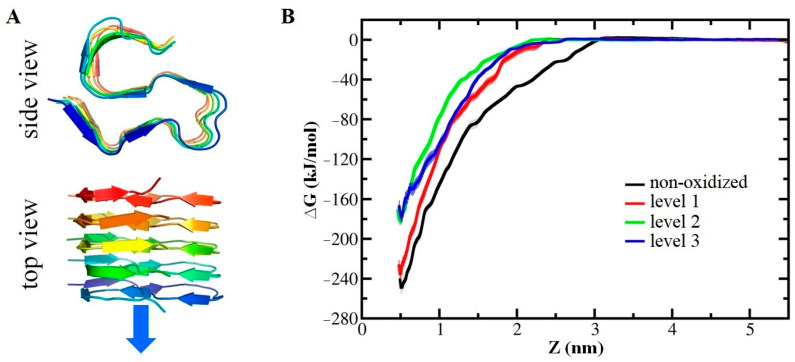
(**A**) Schematic illustration of the Aβ fibril in side and top view. The blue arrow indicates the direction of separation of one chain of the Aβ fibril from the remained chains. (**B**) Free energy profiles of dissociation of one chain from other Aβ chains, where they are both in non-oxidized form (black curve), and at different levels of oxidation (red, green, and blue curves). Reprinted/adapted with permission from Ref. [195]. 2019, The Authors.

CAP also finds application in the treatment of Alzheimer’s disease (AD), a neurodegenerative and progressive disease [322,323]. AD is characterized by symptoms such as dementia, including impaired memory, cognitive decline, and difficulties with motor skills [324]. The accumulation of amyloid beta (Aβ) peptide is a known protein involved in the progression of AD [325,326,327]. Aβ peptide is involved in neural cell function, but its accumulation and dysregulation have been implicated in neural cell death. Aβ peptide is known to contribute to the over-excitatory activity of potassium channels, which can lead to neural apoptosis or programmed cell death [328,329]. In its accumulated and aggregated form (Aβ fibril), Aβ becomes one of the principal toxic species in neural tissues [325]. The aggregated Aβ interacts with the cell membrane, increasing membrane conductance and calcium influx, which can induce cell apoptosis [326,330]. Therefore, targeting the accumulated form of Aβ has been the focus of many studies [331,332], aiming to disaggregate Aβ fibrils or inhibit the accumulation of Aβ peptides. CAP, as a source of RONS, has shown significant effectiveness in reducing Aβ fibrils even after a short exposure time [322,323]. To understand the atomic-level mechanisms of the effect of CAP on Aβ fibrils, it was necessary to investigate the effects of amino acid oxidation on Aβ fibril modifications using MD simulations. A key study by Brown et al. highlighted the role of Met_35_ oxidation in preventing the aggregation of Aβ [333]. Their results demonstrated that oxidation of Met_35_ reduced β-sheet structures, independent of solvent conditions (pH and salt concentration). The reduction of β-sheets is consistent with the experimentally observed reduction in aggregation rate [334]. Building upon this study, Razzokov et al. [195] investigated the stability and disaggregation of Aβ fibrils after the oxidation of Met_35_ at the first level of oxidation, as well as other amino acid residues at subsequent oxidation levels. Their findings showed that increasing oxidation levels resulted in the further breaking of hydrogen bonds and salt bridges, reduction of β-sheets, and destabilization of the system with higher fluctuations. Additionally, the free energy of dissociation between one monomer and other Aβ chains, obtained through US simulations, increased with higher levels of oxidation (see Figure 12). This indicated a reduced attractive interaction between one monomer and other Aβ chains with increased oxidation, which qualitatively aligned with experimental results, showing the potential of CAP-generated RONS oxidation in disaggregating Aβ fibril and potentially treating AD [195,322,323]. Overall, both MD studies concluded that Met_35_ oxidation plays a crucial role in the disaggregation and inhibition of fibril formation of Aβ peptides, contributing to AD treatment.

Apart from membrane and intracellular proteins, other proteins such as mammalian or bacterial lysozyme can also play a therapeutic role in treating specific diseases [335,336,337]. The dynamic nature of cancer cells is primarily responsible for their drug resistance, requiring higher drug doses and resulting in more side effects for patients [338]. Generally, lysozyme is a part of the innate immune system [339] and a natural antibacterial product [340] that can hydrolyze the peptidoglycan linkage in bacterial cell walls [341,342]. It also plays a crucial role in inhibiting tumor formation and growth, increasing the effectiveness of chemotherapy drugs in cancer treatment [340,343]. Therefore, enhancing the efficiency of lysozyme as a cancer therapy drug through structural and functional modifications can be essential in this field. CAP treatment, by oxidizing and nitrating the amino acids of lysozyme, provides a useful method to achieve this goal and design new cancer drugs [196]. Experimental investigations conducted by Attri et al. on the modifications of lysozyme through CAP demonstrated that changes to Trp and Tyr residues of lysozyme by CAP-generated RONS could enhance its activity [196]. Additionally, the study of the structure and dynamics of oxidized lysozyme at acidic and neutral pH through MD simulations indicated that oxidation at neutral pH-maintained stability, while acidic pH increased flexibility and instability, leading to denaturation and subsequent loss of activity [196]. Although this investigation focused on the stability of oxidized lysozyme, further atomic-scale research is required to determine how oxidation enhances its therapeutic activities.

## 4. Conclusions and Perspectives

In this review, we have provided an overview of the current state of non-reactive MD simulations in the field of plasma medicine, specifically focusing on the effect of plasma on biomolecules such as lipid membranes and proteins. This is a highly intricate area of research, and while there have been limited simulation studies conducted so far, several investigations have been carried out for systems and processes closely related to plasma medicine.

To date, atomic scale simulations have primarily concentrated on the nitration and oxidation of lipid membranes, examining their impact on various membrane properties, such as density, thickness, surface area, fluidity, and stiffness, as well as permeability to water, free radicals, and small molecules. These modifications, resulting from RONS generated by CAP, ultimately influence the function of phospholipids and their ability to protect cells against external threats. Additionally, non-reactive MD simulations have explored pore formation in membranes induced by lipid oxidation, which can be further facilitated by external mechanical stress and electric fields, potentially leading to membrane rupture. The increased membrane permeability caused by pore formation can have implications for drug delivery and the treatment of cancer cells. It is crucial to consider factors such as lipid types (e.g., phospholipids and cholesterols), their properties (including polarity, lipid tail type, and head group size), the nature of oxidation and/or nitration, as well as external effects such as electric fields and mechanical stress, as they all significantly contribute to understanding the mechanisms underlying plasma-lipid membrane interactions.

Non-reactive MD simulations have also demonstrated that nitro-oxidation can disrupt the normal functions of membrane proteins, including transmembrane proteins, membrane enzymes, and membrane receptors. Nitro-oxidation of these proteins can affect their structure and function, enzymatic activity, ligand binding, and signaling pathways, offering potential targets for cancer therapy and other diseases. Furthermore, MD simulations have been employed to explore the consequences of nitro-oxidation on intracellular proteins, revealing its influence on the functionality and stability of proteins crucial for cell growth, signaling pathways, and the progression of diseases. The general agreement obtained between MD simulations and experimental findings in numerous studies served as strong evidence that MD simulations are a reliable and cost-effective approach for examining and predicting the effect of CAP on protein structures and, as a result, on cells and tissues. Overall, simulation results have indicated that understanding the structural changes in proteins and alterations in their functions and enzymatic activities due to nitro-oxidation can contribute to the development of new plasma-based therapies targeting these proteins.

MD simulations in the field of plasma medicine are still relatively limited, and further research is necessary to fully understand the mechanisms underlying the effects of CAP on biomolecules. However, with the advancements in force field accuracy and computational power, it is anticipated that new model systems will be investigated, such as the interaction of RNS with various types of proteins, or the study of nitro-oxidation and pore formation in more complex and realistic cell membranes. Simulations are expected to become an indispensable tool for unraveling mechanisms and providing insights into processes in plasma medicine, as their practicality and scope continue to improve.

## References

[1] Chauvin J., Judée F., Yousfi M., Vicendo P., Merbahi N. (2017). Analysis of reactive oxygen and nitrogen species generated in three liquid media by low temperature helium plasma jet. Sci. Rep..

[2] Yan D., Sherman J.H., Keidar M. (2017). Cold atmospheric plasma, a novel promising anti-cancer treatment modality. Oncotarget.

[3] Metelmann H.-R., Seebauer C., Miller V., Fridman A., Bauer G., Graves D.B., Pouvesle J.-M., Rutkowski R., Schuster M., Bekeschus S. (2018). Clinical experience with cold plasma in the treatment of locally advanced head and neck cancer. Clin. Plasma Med..

[4] Ma M., Duan J., Lu X., He G. (2019). Genotoxic and mutagenic properties of atmospheric pressure plasma jet on human liver cell line L02. Phys. Plasmas.

[5] Kubinova S., Zaviskova K., Uherkova L., Zablotskii V., Churpita O., Lunov O., Dejneka A. (2017). Non-thermal air plasma promotes the healing of acute skin wounds in rats. Sci. Rep..

[6] Gao J., Wang L., Xia C., Yang X., Cao Z., Zheng L., Ko R., Shen C., Yang C., Cheng C. (2019). Cold atmospheric plasma promotes different types of superficial skin erosion wounds healing. Int. Wound J..

[7] Daeschlein G., Napp M., Lutze S., Arnold A., von Podewils S., Guembel D., Jünger M. (2015). Skin and wound decontamination of multidrug-resistant bacteria by cold atmospheric plasma coagulation. JDDG J. Der Dtsch. Dermatol. Ges..

[8] Boekema B., Stoop M., Vlig M., van Liempt J., Sobota A., Ulrich M., Middelkoop E. (2021). Antibacterial and safety tests of a flexible cold atmospheric plasma device for the stimulation of wound healing. Appl. Microbiol. Biotechnol..

[9] Liao X., Liu D., Ding T. (2020). Nonthermal plasma induces the viable-but-nonculturable state in Staphylococcus aureus via metabolic suppression and the oxidative stress response. Appl. Environ. Microbiol..

[10] Bernhardt T., Semmler M.L., Schäfer M., Bekeschus S., Emmert S., Boeckmann L. (2019). Plasma medicine: Applications of cold atmospheric pressure plasma in dermatology. Oxidative Med. Cell. Longev..

[11] Gan L., Duan J., Zhang S., Liu X., Poorun D., Liu X., Lu X., Duan X., Liu D., Chen H. (2019). Cold atmospheric plasma ameliorates imiquimod-induced psoriasiform dermatitis in mice by mediating antiproliferative effects. Free Radic. Res..

[12] Fridman G., Peddinghaus M., Balasubramanian M., Ayan H., Fridman A., Gutsol A., Brooks A. (2006). Blood coagulation and living tissue sterilization by floating-electrode dielectric barrier discharge in air. Plasma Chem. Plasma Process..

[13] Lu X., Naidis G.V., Laroussi M., Reuter S., Graves D.B., Ostrikov K. (2016). Reactive species in non-equilibrium atmospheric-pressure plasmas: Generation, transport, and biological effects. Phys. Rep..

[14] Brullé L., Vandamme M., Riès D., Martel E., Robert E., Lerondel S., Trichet V., Richard S., Pouvesle J.-M., Le Pape A. (2012). Effects of a non thermal plasma treatment alone or in combination with gemcitabine in a MIA PaCa2-luc orthotopic pancreatic carcinoma model. PLoS ONE.

[15] Köritzer J., Boxhammer V., Schäfer A., Shimizu T., Klämpfl T.G., Li Y.-F., Welz C., Schwenk-Zieger S., Morfill G.E., Zimmermann J.L. (2013). Restoration of sensitivity in chemo—Resistant glioma cells by cold atmospheric plasma. PLoS ONE.

[16] Ja Kim S., Min Joh H., Chung T.H. (2013). Production of intracellular reactive oxygen species and change of cell viability induced by atmospheric pressure plasma in normal and cancer cells. Appl. Phys. Lett..

[17] Siu A., Volotskova O., Cheng X., Khalsa S.S., Bian K., Murad F., Keidar M., Sherman J.H. (2015). Differential effects of cold atmospheric plasma in the treatment of malignant glioma. PLoS ONE.

[18] Lin A., Gorbanev Y., De Backer J., Van Loenhout J., Van Boxem W., Lemière F., Cos P., Dewilde S., Smits E., Bogaerts A. (2019). Non-thermal plasma as a unique delivery system of short-lived reactive oxygen and nitrogen species for immunogenic cell death in melanoma cells. Adv. Sci..

[19] Kim S.J., Chung T. (2016). Cold atmospheric plasma jet-generated RONS and their selective effects on normal and carcinoma cells. Sci. Rep..

[20] Utsumi F., Kajiyama H., Nakamura K., Tanaka H., Hori M., Kikkawa F. (2014). Selective cytotoxicity of indirect nonequilibrium atmospheric pressure plasma against ovarian clear-cell carcinoma. SpringerPlus.

[21] Zucker S.N., Zirnheld J., Bagati A., DiSanto T.M., Des Soye B., Wawrzyniak J.A., Etemadi K., Nikiforov M., Berezney R. (2012). Preferential induction of apoptotic cell death in melanoma cells as compared with normal keratinocytes using a non-thermal plasma torch. Cancer Biol. Ther..

[22] Ishaq M., Evans M.D., Ostrikov K.K. (2014). Atmospheric pressure gas plasma-induced colorectal cancer cell death is mediated by Nox2–ASK1 apoptosis pathways and oxidative stress is mitigated by Srx–Nrf2 anti-oxidant system. Biochim. Biophys. Acta (BBA)-Mol. Cell Res..

[23] Ishaq M., Kumar S., Varinli H., Han Z.J., Rider A.E., Evans M.D., Murphy A.B., Ostrikov K. (2014). Atmospheric gas plasma–induced ROS production activates TNF-ASK1 pathway for the induction of melanoma cancer cell apoptosis. Mol. Biol. Cell.

[24] Biscop E., Lin A., Van Boxem W., Van Loenhout J., De Backer J., Deben C., Dewilde S., Smits E., Bogaerts A. (2019). The Influence of Cell Type and Culture Medium on Determining Cancer Selectivity of Cold Atmospheric Plasma Treatment. Cancers.

[25] Ratovitski E.A., Cheng X., Yan D., Sherman J.H., Canady J., Trink B., Keidar M. (2014). Anti-cancer therapies of 21st century: Novel approach to treat human cancers using cold atmospheric plasma. Plasma Process. Polym..

[26] Yang H., Lu R., Xian Y., Gan L., Lu X., Yang X. (2015). Effects of atmospheric pressure cold plasma on human hepatocarcinoma cell and its 5-fluorouracil resistant cell line. Phys. Plasmas.

[27] Amini M., Ghanavi J., Farnia P., Karimi M., Ghomi H. (2020). In vitro antiproliferative activity of cold atmospheric plasma on small-cell lung carcinoma. Biomed. Biotechnol. Res. J. (BBRJ).

[28] Sadoughi A., Irani S., Bagheri-Khoulenjani S., Atyabi S.M., Olov N. (2021). Cold atmospheric plasma modification of curcumin loaded in tri-phosphate chitosan nanoparticles enhanced breast cancer cells apoptosis. Polym. Adv. Technol..

[29] Privat-Maldonado A., Gorbanev Y., Dewilde S., Smits E., Bogaerts A. (2018). Reduction of human glioblastoma spheroids using cold atmospheric plasma: The combined effect of short-and long-lived reactive species. Cancers.

[30] Verloy R., Privat-Maldonado A., Smits E., Bogaerts A. (2020). Cold atmospheric plasma treatment for pancreatic cancer–the importance of pancreatic stellate cells. Cancers.

[31] Rutkowski R., Schuster M., Unger J., Seebauer C., Metelmann H., Woedtke T.v., Weltmann K., Daeschlein G. (2017). Hyperspectral imaging for in vivo monitoring of cold atmospheric plasma effects on microcirculation in treatment of head and neck cancer and wound healing. Clin. Plasma Med..

[32] Ozben T. (2007). Oxidative stress and apoptosis: Impact on cancer therapy. J. Pharm. Sci..

[33] Gaur N., Kurita H., Oh J.-S., Miyachika S., Ito M., Mizuno A., Cowin A.J., Allinson S., Short R.D., Szili E.J. (2020). On cold atmospheric-pressure plasma jet induced DNA damage in cells. J. Phys. D Appl. Phys..

[34] Volotskova O., Hawley T.S., Stepp M.A., Keidar M. (2012). Targeting the cancer cell cycle by cold atmospheric plasma. Sci. Rep..

[35] Boeckmann L., Schäfer M., Bernhardt T., Semmler M.L., Jung O., Ojak G., Fischer T., Peters K., Nebe B., Müller-Hilke B. (2020). Cold Atmospheric Pressure Plasma in Wound Healing and Cancer Treatment. Appl. Sci..

[36] Yusupov M., Lackmann J.-W., Razzokov J., Kumar S., Stapelmann K., Bogaerts A. (2018). Impact of plasma oxidation on structural features of human epidermal growth factor. Plasma Process. Polym..

[37] Brehmer F., Haenssle H., Daeschlein G., Ahmed R., Pfeiffer S., Görlitz A., Simon D., Schön M., Wandke D., Emmert S. (2015). Alleviation of chronic venous leg ulcers with a hand-held dielectric barrier discharge plasma generator (PlasmaDerm^®^ VU-2010): Results of a monocentric, two-armed, open, prospective, randomized and controlled trial (NCT 01415622). J. Eur. Acad. Dermatol. Venereol..

[38] Wende K., Straßenburg S., Haertel B., Harms M., Holtz S., Barton A., Masur K., von Woedtke T., Lindequist U. (2014). Atmospheric pressure plasma jet treatment evokes transient oxidative stress in HaCaT keratinocytes and influences cell physiology. Cell Biol. Int..

[39] Dezest M., Chavatte L., Bourdens M., Quinton D., Camus M., Garrigues L., Descargues P., Arbault S., Burlet-Schiltz O., Casteilla L. (2017). Mechanistic insights into the impact of Cold Atmospheric Pressure Plasma on human epithelial cell lines. Sci. Rep..

[40] Min T., Xie X., Ren K., Sun T., Wang H., Dang C., Zhang H. (2022). Therapeutic Effects of Cold Atmospheric Plasma on Solid Tumor. Front. Med..

[41] Gorbanev Y., Privat-Maldonado A., Bogaerts A. (2018). Analysis of short-lived reactive species in plasma–air–water systems: The dos and the do nots. Anal. Chem..

[42] Murillo D., Huergo C., Gallego B., Rodríguez R., Tornín J. (2023). Exploring the Use of Cold Atmospheric Plasma to Overcome Drug Resistance in Cancer. Biomedicines.

[43] Horn A., Jaiswal J.K. (2019). Structural and signaling role of lipids in plasma membrane repair. Curr. Top. Membr..

[44] Watson H. (2015). Biological membranes. Essays Biochem..

[45] Guo Y., Baulin V.A., Thalmann F. (2016). Peroxidised phospholipid bilayers: Insight from coarse-grained molecular dynamics simulations. Soft Matter..

[46] Lis M., Wizert A., Przybylo M., Langner M., Swiatek J., Jungwirth P., Cwiklik L. (2011). The effect of lipid oxidation on the water permeability of phospholipids bilayers. Phys. Chem. Chem. Phys..

[47] Runas K.A., Malmstadt N. (2015). Low levels of lipid oxidation radically increase the passive permeability of lipid bilayers. Soft Matter.

[48] Oliveira M.C., Yusupov M., Cordeiro R.M., Bogaerts A. (2021). Unraveling the permeation of reactive species across nitrated membranes by computer simulations. Comput. Biol. Med..

[49] Van der Paal J., Fridman G., Bogaerts A. (2019). Ceramide cross-linking leads to pore formation: Potential mechanism behind CAP enhancement of transdermal drug delivery. Plasma Process. Polym..

[50] Van der Paal J., Hong S.H., Yusupov M., Gaur N., Oh J.S., Short R.D., Szili E.J., Bogaerts A. (2019). How membrane lipids influence plasma delivery of reactive oxygen species into cells and subsequent DNA damage: An experimental and computational study. Phys. Chem. Chem. Phys..

[51] Bogaerts A., Yusupov M., Razzokov J., Van der Paal J. (2019). Plasma for cancer treatment: How can RONS penetrate through the cell membrane? Answers from computer modeling. Front. Chem. Sci. Eng..

[52] Halliwell B. (1996). Antioxidants in human health and disease. Annu. Rev. Nutr..

[53] Sies H. (2015). Oxidative stress: A concept in redox biology and medicine. Redox Biol..

[54] Shaw P., Kumar N., Sahun M., Smits E., Bogaerts A., Privat-Maldonado A. (2022). Modulating the Antioxidant Response for Better Oxidative Stress-Inducing Therapies: How to Take Advantage of Two Sides of the Same Medal?. Biomedicines.

[55] Bauer G. (2014). Targeting extracellular ROS signaling of tumor cells. Anticancer Res..

[56] Bauer G. (2015). Increasing the endogenous NO level causes catalase inactivation and reactivation of intercellular apoptosis signaling specifically in tumor cells. Redox Biol..

[57] Bauer G. (2016). Nitric oxide’s contribution to selective apoptosis induction in malignant cells through multiple reaction steps. Crit. Rev. Oncog..

[58] Almén M.S., Nordström K.J., Fredriksson R., Schiöth H.B. (2009). Mapping the human membrane proteome: A majority of the human membrane proteins can be classified according to function and evolutionary origin. BMC Biol..

[59] Van der Paal J., Neyts E.C., Verlackt C.C., Bogaerts A. (2016). Effect of lipid peroxidation on membrane permeability of cancer and normal cells subjected to oxidative stress. Chem. Sci..

[60] Bogaerts A., Khosravian N., Van der Paal J., Verlackt C.C., Yusupov M., Kamaraj B., Neyts E.C. (2015). Multi-level molecular modelling for plasma medicine. J. Phys. D Appl. Phys..

[61] Laroussi M., Bekeschus S., Keidar M., Bogaerts A., Fridman A., Lu X., Ostrikov K., Hori M., Stapelmann K., Miller V. (2022). Low-temperature plasma for biology, hygiene, and medicine: Perspective and roadmap. IEEE Trans. Radiat. Plasma Med. Sci..

[62] Isbary G., Shimizu T., Li Y.-F., Stolz W., Thomas H.M., Morfill G.E., Zimmermann J.L. (2013). Cold atmospheric plasma devices for medical issues. Expert Rev. Med. Devices.

[63] Yusupov M., Neyts E., Simon P., Berdiyorov G., Snoeckx R., Van Duin A., Bogaerts A. (2013). Reactive molecular dynamics simulations of oxygen species in a liquid water layer of interest for plasma medicine. J. Phys. D Appl. Phys..

[64] Knight C., Lindberg G.E., Voth G.A. (2012). Multiscale reactive molecular dynamics. J. Chem. Phys..

[65] Bogaerts A., Yusupov M., Van der Paal J., Verlackt C.C., Neyts E.C. (2014). Reactive molecular dynamics simulations for a better insight in plasma medicine. Plasma Process. Polym..

[66] Dror R.O., Dirks R.M., Grossman J., Xu H., Shaw D.E. (2012). Biomolecular simulation: A computational microscope for molecular biology. Annu. Rev. Biophys..

[67] Nair P.C., Miners J.O. (2014). Molecular dynamics simulations: From structure function relationships to drug discovery. Silico Pharmacol..

[68] May A., Pool R., van Dijk E., Bijlard J., Abeln S., Heringa J., Feenstra K.A. (2014). Coarse-grained versus atomistic simulations: Realistic interaction free energies for real proteins. Bioinformatics.

[69] Monticelli L., Tieleman D.P. (2013). Force fields for classical molecular dynamics. Biomol. Simul. Methods Protoc..

[70] Van Meer G., Voelker D.R., Feigenson G.W. (2008). Membrane lipids: Where they are and how they behave. Nat. Rev. Mol. Cell Biol..

[71] Oliveira M.C., Yusupov M., Bogaerts A., Cordeiro R.M. (2020). How do nitrated lipids affect the properties of phospholipid membranes?. Arch. Biochem. Biophys..

[72] Oliveira M.C., Yusupov M., Bogaerts A., Cordeiro R.M. (2019). Molecular dynamics simulations of mechanical stress on oxidized membranes. Biophys. Chem..

[73] Oliveira M.C., Yusupov M., Bogaerts A., Cordeiro R.M. (2021). Lipid Oxidation: Role of Membrane Phase-Separated Domains. J. Chem. Inf. Model..

[74] Oliveira M.C., Yusupov M., Bogaerts A., Cordeiro R.M. (2022). Distribution of lipid aldehydes in phase-separated membranes: A molecular dynamics study. Arch. Biochem. Biophys..

[75] Yusupov M., Van der Paal J., Neyts E.C., Bogaerts A. (2017). Synergistic effect of electric field and lipid oxidation on the permeability of cell membranes. Biochim. Biophys. Acta Gen. Subj..

[76] Yusupov M., Wende K., Kupsch S., Neyts E.C., Reuter S., Bogaerts A. (2017). Effect of head group and lipid tail oxidation in the cell membrane revealed through integrated simulations and experiments. Sci. Rep..

[77] Plaa G.L., Witschi H. (1976). Chemicals, drugs, and lipid peroxidation. Annu. Rev. Pharmacol. Toxicol..

[78] Naito Y., Yoshikawa T., Yoshida N., Kondo M. (1998). Role of oxygen radical and lipid peroxidation in indomethacin-induced gastric mucosal injury. Dig. Dis. Sci..

[79] Ye L.F., Chaudhary K.R., Zandkarimi F., Harken A.D., Kinslow C.J., Upadhyayula P.S., Dovas A., Higgins D.M., Tan H., Zhang Y. (2020). Radiation-induced lipid peroxidation triggers ferroptosis and synergizes with ferroptosis inducers. ACS Chem. Biol..

[80] Yusupov M., Razzokov J., Cordeiro R.M., Bogaerts A. (2019). Transport of Reactive Oxygen and Nitrogen Species across Aquaporin: A Molecular Level Picture. Oxid. Med. Cell Longev..

[81] Razzokov J., Yusupov M., Cordeiro R.M., Bogaerts A. (2018). Atomic scale understanding of the permeation of plasma species across native and oxidized membranes. J. Phys. D Appl. Phys..

[82] Hermetter A., Kopec W., Khandelia H. (2013). Conformations of double-headed, triple-tailed phospholipid oxidation lipid products in model membranes. Biochim. Biophys. Acta.

[83] Yin H., Xu L., Porter N.A. (2011). Free radical lipid peroxidation: Mechanisms and analysis. Chem. Rev..

[84] Jurkiewicz P., Olżyńska A., Cwiklik L., Conte E., Jungwirth P., Megli F.M., Hof M. (2012). Biophysics of lipid bilayers containing oxidatively modified phospholipids: Insights from fluorescence and EPR experiments and from MD simulations. Biochim. Biophys. Acta (BBA)-Biomembr..

[85] Lee H., Malmstadt N. (2018). Effect of low levels of lipid oxidation on the curvature, dynamics, and permeability of lipid bilayers and their interactions with cationic nanoparticles. J. Phys. D Appl. Phys..

[86] Aceves-Luna H., Glossman-Mitnik D., Flores-Holguin N. (2022). Oxidation degree of a cell membrane model and its response to structural changes, a coarse-grained molecular dynamics approach. J. Biomol. Struct. Dyn..

[87] Agmon E., Solon J., Bassereau P., Stockwell B.R. (2018). Modeling the effects of lipid peroxidation during ferroptosis on membrane properties. Sci. Rep..

[88] Donner M., Muller S., Stoltz J. (1990). Importance of membrane fluidity determination. J. Des. Mal. Vasc..

[89] Bernardes N., Fialho A.M. (2018). Perturbing the dynamics and organization of cell membrane components: A new paradigm for cancer-targeted therapies. Int. J. Mol. Sci..

[90] Casares D., Escribá P.V., Rosselló C.A. (2019). Membrane lipid composition: Effect on membrane and organelle structure, function and compartmentalization and therapeutic avenues. Int. J. Mol. Sci..

[91] Pakiet A., Kobiela J., Stepnowski P., Sledzinski T., Mika A. (2019). Changes in lipids composition and metabolism in colorectal cancer: A review. Lipids Health Dis..

[92] Szlasa W., Zendran I., Zalesińska A., Tarek M., Kulbacka J. (2020). Lipid composition of the cancer cell membrane. J. Bioenerg. Biomembr..

[93] Zalba S., Ten Hagen T.L. (2017). Cell membrane modulation as adjuvant in cancer therapy. Cancer Treat. Rev..

[94] Preta G. (2020). New insights into targeting membrane lipids for cancer therapy. Front. Cell Dev. Biol..

[95] Li C., Zhang G., Zhao L., Ma Z., Chen H. (2015). Metabolic reprogramming in cancer cells: Glycolysis, glutaminolysis, and Bcl-2 proteins as novel therapeutic targets for cancer. World J. Surg. Oncol..

[96] Cazzola R., Rondanelli M., Russo-Volpe S., Ferrari E., Cestaro B. (2004). Decreased membrane fluidity and altered susceptibility to peroxidation and lipid composition in overweight and obese female erythrocytes. J. Lipid Res..

[97] Ayee M.A.A., LeMaster E., Shentu T.P., Singh D.K., Barbera N., Soni D., Tiruppathi C., Subbaiah P.V., Berdyshev E., Bronova I. (2017). Molecular-Scale Biophysical Modulation of an Endothelial Membrane by Oxidized Phospholipids. Biophys. J..

[98] Kumar S., Rana R., Yadav D.K. (2021). Atomic-scale modeling of the effect of lipid peroxidation on the permeability of reactive species. J. Biomol. Struct. Dyn..

[99] Ingólfsson H.I., Melo M.N., Van Eerden F.J., Arnarez C., Lopez C.A., Wassenaar T.A., Periole X., De Vries A.H., Tieleman D.P., Marrink S.J. (2014). Lipid organization of the plasma membrane. J. Am. Chem. Soc..

[100] Jarerattanachat V., Karttunen M., Wong-Ekkabut J. (2013). Molecular dynamics study of oxidized lipid bilayers in NaCl solution. J. Phys. Chem. B.

[101] Moore K., Roberts L.J. (1998). Measurement of lipid peroxidation. Free Radic. Res..

[102] Uchida K. (2000). Role of reactive aldehyde in cardiovascular diseases. Free Radic. Biol. Med..

[103] Plochberger B., Stockner T., Chiantia S., Brameshuber M., Weghuber J., Hermetter A., Schwille P., Schütz G.J. (2010). Cholesterol slows down the lateral mobility of an oxidized phospholipid in a supported lipid bilayer. Langmuir.

[104] Wong-Ekkabut J., Xu Z., Triampo W., Tang I.M., Tieleman D.P., Monticelli L. (2007). Effect of lipid peroxidation on the properties of lipid bilayers: A molecular dynamics study. Biophys. J..

[105] Beranova L., Cwiklik L., Jurkiewicz P., Hof M., Jungwirth P. (2010). Oxidation changes physical properties of phospholipid bilayers: Fluorescence spectroscopy and molecular simulations. Langmuir.

[106] Schumann-Gillett A., O’Mara M.L. (2019). The effects of oxidised phospholipids and cholesterol on the biophysical properties of POPC bilayers. Biochim. Biophys. Acta Biomembr..

[107] Loura L.M., do Canto A.M.M., Martins J. (2013). Sensing hydration and behavior of pyrene in POPC and POPC/cholesterol bilayers: A molecular dynamics study. Biochim. Biophys. Acta (BBA)-Biomembr..

[108] Alberts B., Johnson A., Lewis J., Raff M., Roberts K., Walter P. (2008). Molecular Biology of the Cell.

[109] Bompard J., Rosso A., Brizuela L., Mebarek S., Blum L.J., Trunfio-Sfarghiu A.M., Lollo G., Granjon T., Girard-Egrot A., Maniti O. (2020). Membrane Fluidity as a New Means to Selectively Target Cancer Cells with Fusogenic Lipid Carriers. Langmuir.

[110] Bunker A., Magarkar A., Viitala T. (2016). Rational design of liposomal drug delivery systems, a review: Combined experimental and computational studies of lipid membranes, liposomes and their PEGylation. Biochim. Biophys. Acta (BBA)-Biomembr..

[111] Dhawan V., Magarkar A., Joshi G., Makhija D., Jain A., Shah J., Reddy B., Krishnapriya M., Róg T., Bunker A. (2016). Stearylated cycloarginine nanosystems for intracellular delivery–simulations, formulation and proof of concept. RSC Adv..

[112] Pathak P., Dhawan V., Magarkar A., Danne R., Govindarajan S., Ghosh S., Steiniger F., Chaudhari P., Gopal V., Bunker A. (2016). Design of cholesterol arabinogalactan anchored liposomes for asialoglycoprotein receptor mediated targeting to hepatocellular carcinoma: In silico modeling, in vitro and in vivo evaluation. Int. J. Pharm..

[113] Olzynska A., Kulig W., Mikkolainen H., Czerniak T., Jurkiewicz P., Cwiklik L., Rog T., Hof M., Jungwirth P., Vattulainen I. (2020). Tail-Oxidized Cholesterol Enhances Membrane Permeability for Small Solutes. Langmuir.

[114] Khandelia H., Loubet B., Olzynska A., Jurkiewicz P., Hof M. (2014). Pairing of cholesterol with oxidized phospholipid species in lipid bilayers. Soft Matter.

[115] Kulig W., Olzynska A., Jurkiewicz P., Kantola A.M., Komulainen S., Manna M., Pourmousa M., Vazdar M., Cwiklik L., Rog T. (2015). Cholesterol under oxidative stress-How lipid membranes sense oxidation as cholesterol is being replaced by oxysterols. Free Radic. Biol. Med..

[116] Hu Y., Zhao T., Zou L., Wang X., Zhang Y. (2019). Molecular dynamics simulations of membrane properties affected by plasma ROS based on the GROMOS force field. Biophys. Chem..

[117] Umetani M., Domoto H., Gormley A.K., Yuhanna I.S., Cummins C.L., Javitt N.B., Korach K.S., Shaul P.W., Mangelsdorf D.J. (2007). 27-Hydroxycholesterol is an endogenous SERM that inhibits the cardiovascular effects of estrogen. Nat. Med..

[118] Murdolo G., Bartolini D., Tortoioli C., Piroddi M., Iuliano L., Galli F. (2013). Lipokines and oxysterols: Novel adipose-derived lipid hormones linking adipose dysfunction and insulin resistance. Free Radic. Biol. Med..

[119] Gosselet F., Saint-Pol J., Fenart L. (2014). Effects of oxysterols on the blood–brain barrier: Implications for Alzheimer’s disease. Biochem. Biophys. Res. Commun..

[120] Kölsch H., Lütjohann D., Von Bergmann K., Heun R. (2003). The role of 24S-hydroxycholesterol in Alzheimer’s disease. J. Nutr. Health Aging.

[121] Kulig W., Cwiklik L., Jurkiewicz P., Rog T., Vattulainen I. (2016). Cholesterol oxidation products and their biological importance. Chem. Phys. Lipids.

[122] Wilson K.A., Wang L., O’Mara M.L. (2021). Site of Cholesterol Oxidation Impacts Its Localization and Domain Formation in the Neuronal Plasma Membrane. ACS Chem. Neurosci..

[123] Neto A.J.P., Cordeiro R.M. (2016). Molecular simulations of the effects of phospholipid and cholesterol peroxidation on lipid membrane properties. Biochim. Biophys. Acta.

[124] Lei K., Kurum A., Kaynak M., Bonati L., Han Y., Cencen V., Gao M., Xie Y.-Q., Guo Y., Hannebelle M.T. (2021). Cancer-cell stiffening via cholesterol depletion enhances adoptive T-cell immunotherapy. Nat. Biomed. Eng..

[125] Sok M., Šentjurc M., Schara M., Stare J., Rott T. (2002). Cell membrane fluidity and prognosis of lung cancer. Ann. Thorac. Surg..

[126] Zeisig R., Koklič T., Wiesner B., Fichtner I., Sentjurč M. (2007). Increase in fluidity in the membrane of MT3 breast cancer cells correlates with enhanced cell adhesion in vitro and increased lung metastasis in NOD/SCID mice. Arch. Biochem. Biophys..

[127] Peetla C., Vijayaraghavalu S., Labhasetwar V. (2013). Biophysics of cell membrane lipids in cancer drug resistance: Implications for drug transport and drug delivery with nanoparticles. Adv. Drug Deliv. Rev..

[128] Maja M., Tyteca D. (2022). Alteration of cholesterol distribution at the plasma membrane of cancer cells: From evidence to pathophysiological implication and promising therapy strategy. Front. Physiol..

[129] Dobretsov G., Borschevskaya T., Petrov V., Vladimirov Y.A. (1977). The increase of phospholipid bilayer rigidity after lipid peroxidation. FEBS Lett..

[130] Chng C.P., Sadovsky Y., Hsia K.J., Huang C. (2021). Site-Specific Peroxidation Modulates Lipid Bilayer Mechanics. Extrem. Mech. Lett..

[131] Fettiplace R., Haydon D. (1980). Water permeability of lipid membranes. Physiol. Rev..

[132] Razzokov J., Yusupov M., Bogaerts A. (2018). Possible Mechanism of Glucose Uptake Enhanced by Cold Atmospheric Plasma: Atomic Scale Simulations. Plasma.

[133] Frallicciardi J., Melcr J., Siginou P., Marrink S.J., Poolman B. (2022). Membrane thickness, lipid phase and sterol type are determining factors in the permeability of membranes to small solutes. Nat. Commun..

[134] Rems L., Viano M., Kasimova M.A., Miklavčič D., Tarek M. (2019). The contribution of lipid peroxidation to membrane permeability in electropermeabilization: A molecular dynamics study. Bioelectrochemistry.

[135] Moradi S., Nowroozi A., Shahlaei M. (2019). Shedding light on the structural properties of lipid bilayers using molecular dynamics simulation: A review study. RSC Adv..

[136] Siani P., de Souza R.M., Dias L.G., Itri R., Khandelia H. (2016). An overview of molecular dynamics simulations of oxidized lipid systems, with a comparison of ELBA and MARTINI force fields for coarse grained lipid simulations. Biochim. Biophys. Acta.

[137] Khabiri M., Roeselova M., Cwiklik L. (2012). Properties of oxidized phospholipid monolayers: An atomistic molecular dynamics study. Chem. Phys. Lett..

[138] Singh G., Chamberlin A.C., Zhekova H.R., Noskov S.Y., Tieleman D.P. (2016). Two-dimensional potentials of mean force of Nile red in intact and damaged model bilayers. Application to calculations of fluorescence spectra. J. Chem. Theory Comput..

[139] Boonnoy P., Jarerattanachat V., Karttunen M., Wong-Ekkabut J. (2015). Bilayer deformation, pores, and micellation induced by oxidized lipids. J. Phys. Chem. Lett..

[140] Tieleman D.P. (2004). The molecular basis of electroporation. BMC Biochem..

[141] Tieleman D.P., Leontiadou H., Mark A.E., Marrink S.-J. (2003). Simulation of pore formation in lipid bilayers by mechanical stress and electric fields. J. Am. Chem. Soc..

[142] Kotnik T., Rems L., Tarek M., Miklavčič D. (2019). Membrane electroporation and electropermeabilization: Mechanisms and models. Annu. Rev. Biophys..

[143] Gurtovenko A.A., Vattulainen I. (2007). Ion leakage through transient water pores in protein-free lipid membranes driven by transmembrane ionic charge imbalance. Biophys. J..

[144] Marrink S., de Vries A., Tieleman D. (2009). Lipids on the move: Computer simulations of bilayer deformations. Biochim. Biophys. Acta Biomembr..

[145] Lipkin R., Lazaridis T. (2017). Computational studies of peptide-induced membrane pore formation. Philos. Trans. R. Soc. B Biol. Sci..

[146] Manna M., Mukhopadhyay C. (2009). Cause and effect of melittin-induced pore formation: A computational approach. Langmuir.

[147] Cwiklik L., Jungwirth P. (2010). Massive oxidation of phospholipid membranes leads to pore creation and bilayer disintegration. Chem. Phys. Lett..

[148] Volinsky R., Cwiklik L., Jurkiewicz P., Hof M., Jungwirth P., Kinnunen P.K. (2011). Oxidized phosphatidylcholines facilitate phospholipid flip-flop in liposomes. Biophys. J..

[149] Stefl M., Sachl R., Olzynska A., Amaro M., Savchenko D., Deyneka A., Hermetter A., Cwiklik L., Humpolickova J., Hof M. (2014). Comprehensive portrait of cholesterol containing oxidized membrane. Biochim. Biophys. Acta.

[150] Wiczew D., Szulc N., Tarek M. (2021). Molecular dynamics simulations of the effects of lipid oxidation on the permeability of cell membranes. Bioelectrochemistry.

[151] Boonnoy P., Jarerattanachat V., Karttunen M., Wong-Ekkabut J. (2021). Role of cholesterol flip-flop in oxidized lipid bilayers. Biophys. J..

[152] Van der Paal J., Verheyen C., Neyts E.C., Bogaerts A. (2017). Hampering effect of cholesterol on the permeation of reactive oxygen species through phospholipids bilayer: Possible explanation for plasma cancer selectivity. Sci. Rep..

[153] ARENDS M.J., WYLLIE A.H. (1991). Apoptosis: Mechanisms and roles in pathology. Int. Rev. Exp. Pathol..

[154] Zhang Y., Chen X., Gueydan C., Han J. (2018). Plasma membrane changes during programmed cell deaths. Cell Res..

[155] Needham D., Nunn R.S. (1990). Elastic deformation and failure of lipid bilayer membranes containing cholesterol. Biophys. J..

[156] Shigematsu T., Koshiyama K., Wada S. (2015). Effects of stretching speed on mechanical rupture of phospholipid/cholesterol bilayers: Molecular dynamics simulation. Sci. Rep..

[157] Yadav D.K., Kumar S., Choi E.H., Kim M.H. (2021). Electric-field-induced electroporation and permeation of reactive oxygen species across a skin membrane. J. Biomol. Struct. Dyn..

[158] Vernier P.T., Levine Z.A., Wu Y.-H., Joubert V., Ziegler M.J., Mir L.M., Tieleman D.P. (2009). Electroporating fields target oxidatively damaged areas in the cell membrane. PLoS ONE.

[159] Wang H., Zhao T., Wang Z., Wang X., Wang D., Zhang Y. (2023). Molecular dynamics simulations of cancer cell membrane electroporation under the plasma electric field effect. Plasma Process. Polym..

[160] Cheng X., Murphy W., Recek N., Yan D., Cvelbar U., Vesel A., Mozetič M., Canady J., Keidar M., Sherman J.H. (2014). Synergistic effect of gold nanoparticles and cold plasma on glioblastoma cancer therapy. J. Phys. D Appl. Phys..

[161] Irani S., Shahmirani Z., Atyabi S.M., Mirpoor S. (2015). Induction of growth arrest in colorectal cancer cells by cold plasma and gold nanoparticles. Arch. Med. Sci..

[162] Yang H., Li H., Liu L., Zhou Y., Long X. (2019). Molecular simulation studies on the interactions of 2, 4, 6-trinitrotoluene and its metabolites with lipid membranes. J. Phys. Chem. B.

[163] Yang H., Zhou M., Li H., Wei T., Tang C., Zhou Y., Long X. (2020). Effects of Low-level Lipid Peroxidation on the Permeability of Nitroaromatic Molecules across a Membrane: A Computational Study. ACS Omega.

[164] Shaw P., Kumar N., Hammerschmid D., Privat-Maldonado A., Dewilde S., Bogaerts A. (2019). Synergistic Effects of Melittin and Plasma Treatment: A Promising Approach for Cancer Therapy. Cancers.

[165] Fernandez M., Marin R., Proverbio F., Ruette F. (2021). Effect of magnesium sulfate in oxidized lipid bilayers properties by using molecular dynamics. Biochem. Biophys. Rep..

[166] Haralambiev L., Nitsch A., Einenkel R., Muzzio D.O., Gelbrich N., Burchardt M., Zygmunt M., Ekkernkamp A., Stope M.B., Guembel D. (2020). The effect of cold atmospheric plasma on the membrane permeability of human osteosarcoma cells. Anticancer. Res..

[167] Haralambiev L., Nitsch A., Jacoby J.M., Strakeljahn S., Bekeschus S., Mustea A., Ekkernkamp A., Stope M.B. (2020). Cold atmospheric plasma treatment of chondrosarcoma cells affects proliferation and cell membrane permeability. Int. J. Mol. Sci..

[168] Harding C.R. (2004). The stratum corneum: Structure and function in health and disease. Dermatol. Ther..

[169] Van Smeden J., Bouwstra J.A. (2016). Stratum corneum lipids: Their role for the skin barrier function in healthy subjects and atopic dermatitis patients. Ski. Barrier Funct..

[170] Menon G.K., Cleary G.W., Lane M.E. (2012). The structure and function of the stratum corneum. Int. J. Pharm..

[171] Duan J., Ma M., Yusupov M., Cordeiro R.M., Lu X., Bogaerts A. (2020). The penetration of reactive oxygen and nitrogen species across the stratum corneum. Plasma Process. Polym..

[172] Yadav D.K., Kumar S., Choi E.H., Chaudhary S., Kim M.H. (2019). Molecular dynamic simulations of oxidized skin lipid bilayer and permeability of reactive oxygen species. Sci. Rep..

[173] Lu X., Keidar M., Laroussi M., Choi E., Szili E.J., Ostrikov K. (2019). Transcutaneous plasma stress: From soft-matter models to living tissues. Mater. Sci. Eng. R Rep..

[174] Bold R.J., Termuhlen P.M., McConkey D.J. (1997). Apoptosis, cancer and cancer therapy. Surg. Oncol..

[175] Segawa K., Nagata S. (2015). An apoptotic ‘eat me’signal: Phosphatidylserine exposure. Trends Cell Biol..

[176] Schlegel R., Williamson P. (2001). Phosphatidylserine, a death knell. Cell Death Differ..

[177] Armstrong V.T., Brzustowicz M.R., Wassall S.R., Jenski L.J., Stillwell W. (2003). Rapid flip-flop in polyunsaturated (docosahexaenoate) phospholipid membranes. Arch. Biochem. Biophys..

[178] Razzokov J., Yusupov M., Vanuytsel S., Neyts E.C., Bogaerts A. (2017). Phosphatidylserine flip-flop induced by oxidation of the plasma membrane: A better insight by atomic scale modeling. Plasma Process. Polym..

[179] Zarrinnahad H., Mahmoodzadeh A., Hamidi M.P., Mahdavi M., Moradi A., Bagheri K.P., Shahbazzadeh D. (2018). Apoptotic effect of melittin purified from Iranian honey bee venom on human cervical cancer HeLa cell line. Int. J. Pept. Res. Ther..

[180] Maher S., McClean S. (2008). Melittin exhibits necrotic cytotoxicity in gastrointestinal cells which is attenuated by cholesterol. Biochem. Pharmacol..

[181] Shinitzky M. (1984). Membrane fluidity in malignancy adversative and recuperative. Biochim. Biophys. Acta (BBA)-Rev. Cancer.

[182] Van Blitterswijk W., De Veer G., Krol J., Emmelot P. (1982). Comparative lipid analysis of purified plasma membranes and shed extracellular membrane vesicles from normal murine thymocytes and leukemic GRSL cells. Biochim. Biophys. Acta (BBA)-Biomembr..

[183] Harayama T., Riezman H. (2018). Understanding the diversity of membrane lipid composition. Nat. Rev. Mol. Cell Biol..

[184] Cordeiro R.M. (2014). Reactive oxygen species at phospholipid bilayers: Distribution, mobility and permeation. Biochim. Biophys. Acta (BBA)-Biomembr..

[185] Abduvokhidov D., Yusupov M., Shahzad A., Attri P., Shiratani M., Oliveira M.C., Razzokov J. (2023). Unraveling the Transport Properties of RONS across Nitro-Oxidized Membranes. Biomolecules.

[186] Torabizadeh H. (2011). All proteins have a basic molecular formula. World Acad. Sci. Eng. Technol..

[187] Nelson D.L., Lehninger A.L., Cox M.M. (2008). Lehninger Principles of Biochemistry.

[188] Lesk A., Chothia C. (1986). The response of protein structures to amino-acid sequence changes. Philos. Trans. R. Soc. Lond. Ser. A Math. Phys. Sci..

[189] Ghasemitarei M., Yusupov M., Razzokov J., Shokri B., Bogaerts A. (2019). Transport of cystine across xC^−^ antiporter. Arch. Biochem. Biophys..

[190] Ghasemitarei M., Yusupov M., Razzokov J., Shokri B., Bogaerts A. (2019). Effect of oxidative stress on cystine transportation by xC^−^ antiporter. Arch. Biochem. Biophys..

[191] De Backer J., Razzokov J., Hammerschmid D., Mensch C., Hafideddine Z., Kumar N., van Raemdonck G., Yusupov M., Van Doorslaer S., Johannessen C. (2018). The effect of reactive oxygen and nitrogen species on the structure of cytoglobin: A potential tumor suppressor. Redox Biol..

[192] Lin A., Razzokov J., Verswyvel H., Privat-Maldonado A., De Backer J., Yusupov M., Cardenas De La Hoz E., Ponsaerts P., Smits E., Bogaerts A. (2021). Oxidation of Innate Immune Checkpoint CD47 on Cancer Cells with Non-Thermal Plasma. Cancers.

[193] Lin L., Wang L., Liu Y., Xu C., Tu Y., Zhou J. (2018). Non-thermal plasma inhibits tumor growth and proliferation and enhances the sensitivity to radiation in vitro and in vivo. Oncol. Rep..

[194] Razzokov J., Fazliev S., Erkinova D., Mamatkulov S., Chen Z. (2022). Understanding the effect of nitrosylation on dynamics of human epidermal growth factor: A µs simulation study. J. Phys. D Appl. Phys..

[195] Razzokov J., Yusupov M., Bogaerts A. (2019). Oxidation destabilizes toxic amyloid beta peptide aggregation. Sci. Rep..

[196] Attri P., Kaushik N.K., Kaushik N., Hammerschmid D., Privat-Maldonado A., De Backer J., Shiratani M., Choi E.H., Bogaerts A. (2021). Plasma treatment causes structural modifications in lysozyme, and increases cytotoxicity towards cancer cells. Int. J. Biol. Macromol..

[197] Berlett B.S., Stadtman E.R. (1997). Protein oxidation in aging, disease, and oxidative stress. J. Biol. Chem..

[198] Attri P., Park J.H., De Backer J., Kim M., Yun J.H., Heo Y., Dewilde S., Shiratani M., Choi E.H., Lee W. (2020). Structural modification of NADPH oxidase activator (Noxa 1) by oxidative stress: An experimental and computational study. Int. J. Biol. Macromol..

[199] Yusupov M., Privat-Maldonado A., Cordeiro R.M., Verswyvel H., Shaw P., Razzokov J., Smits E., Bogaerts A. (2021). Oxidative damage to hyaluronan-CD44 interactions as an underlying mechanism of action of oxidative stress-inducing cancer therapy. Redox Biol..

[200] Razzokov J., Fazliev S., Yusupov M., Sharipov A., Ruziev Z., Mamatkulov S. (2021). Effect of mutation and disulfide bond formation on the catalytic site of monomeric cytoglobin: A molecular level insight. Plasma Med..

[201] Han I., Song I.S., Choi S.A., Lee T., Yusupov M., Shaw P., Bogaerts A., Choi E.H., Ryu J.J. (2023). Bioactive Nonthermal Biocompatible Plasma Enhances Migration on Human Gingival Fibroblasts. Adv. Heal. Mater..

[202] Attri P., Kurita H., Koga K., Shiratani M. (2021). Impact of Reactive Oxygen and Nitrogen Species Produced by Plasma on Mdm2-p53 Complex. Int. J. Mol. Sci..

[203] Attri P., Koga K., Shiratani M. (2021). Possible impact of plasma oxidation on the structure of the C-terminal domain of SARS-CoV-2 spike protein: A computational study. Appl. Phys. Express.

[204] Ghasemitarei M., Privat-Maldonado A., Yusupov M., Rahnama S., Bogaerts A., Ejtehadi M.R. (2022). Effect of Cysteine Oxidation in SARS-CoV-2 Receptor-Binding Domain on Its Interaction with Two Cell Receptors: Insights from Atomistic Simulations. J. Chem. Inf. Model..

[205] Attri P., Han J., Choi S., Choi E.H., Bogaerts A., Lee W. (2018). CAP modifies the structure of a model protein from thermophilic bacteria: Mechanisms of CAP-mediated inactivation. Sci. Rep..

[206] Takai E., Kitamura T., Kuwabara J., Ikawa S., Yoshizawa S., Shiraki K., Kawasaki H., Arakawa R., Kitano K. (2014). Chemical modification of amino acids by atmospheric-pressure cold plasma in aqueous solution. J. Phys. D Appl. Phys..

[207] Zhou R., Zhou R., Zhuang J., Zong Z., Zhang X., Liu D., Bazaka K., Ostrikov K. (2016). Interaction of Atmospheric-Pressure Air Microplasmas with Amino Acids as Fundamental Processes in Aqueous Solution. PLoS ONE.

[208] Wenske S., Lackmann J.-W., Bekeschus S., Weltmann K.-D., von Woedtke T., Wende K. (2020). Nonenzymatic post-translational modifications in peptides by cold plasma-derived reactive oxygen and nitrogen species. Biointerphases.

[209] Wenske S., Lackmann J.-W., Busch L.M., Bekeschus S., von Woedtke T., Wende K. (2021). Reactive species driven oxidative modifications of peptides—Tracing physical plasma liquid chemistry. J. Appl. Phys..

[210] Alvarez B., Radi R. (2003). Peroxynitrite reactivity with amino acids and proteins. Amino Acids.

[211] Nicolau Jr D.V., Burrage K., Parton R.G., Hancock J.F. (2006). Identifying optimal lipid raft characteristics required to promote nanoscale protein-protein interactions on the plasma membrane. Mol. Cell. Biol..

[212] Yan D., Xiao H., Zhu W., Nourmohammadi N., Zhang L.G., Bian K., Keidar M. (2017). The role of aquaporins in the anti-glioblastoma capacity of the cold plasma-stimulated medium. J. Phys. D Appl. Phys..

[213] Jung H.J., Park J.-Y., Jeon H.-S., Kwon T.-H. (2011). Aquaporin-5: A marker protein for proliferation and migration of human breast cancer cells. PLoS ONE.

[214] Traberg-Nyborg L., Login F.H., Edamana S., Tramm T., Borgquist S., Nejsum L.N. (2022). Aquaporin-1 in breast cancer. APMIS.

[215] Zhang Z., Chen Z., Song Y., Zhang P., Hu J., Bai C. (2010). Expression of aquaporin 5 increases proliferation and metastasis potential of lung cancer. J. Pathol..

[216] Frede J., Fraser S.P., Oskay-Özcelik G., Hong Y., Braicu E.I., Sehouli J., Gabra H., Djamgoz M.B. (2013). Ovarian cancer: Ion channel and aquaporin expression as novel targets of clinical potential. Eur. J. Cancer.

[217] Yan C., Yang J., Shen L., Chen X. (2012). Inhibitory effect of Epigallocatechin gallate on ovarian cancer cell proliferation associated with aquaporin 5 expression. Arch. Gynecol. Obstet..

[218] Wang W., Li Q., Yang T., Li D., Ding F., Sun H., Bai G. (2018). Anti-cancer effect of Aquaporin 5 silencing in colorectal cancer cells in association with inhibition of Wnt/β-catenin pathway. Cytotechnology.

[219] Moon C., Soria J.-C., Jang S.J., Lee J., Hoque M.O., Sibony M., Trink B., Chang Y.S., Sidransky D., Mao L. (2003). Involvement of aquaporins in colorectal carcinogenesis. Oncogene.

[220] Graves D.B. (2014). Reactive species from cold atmospheric plasma: Implications for cancer therapy. Plasma Process. Polym..

[221] Yan D., Talbot A., Nourmohammadi N., Sherman J.H., Cheng X., Keidar M. (2015). Toward understanding the selective anticancer capacity of cold atmospheric plasma—A model based on aquaporins. Biointerphases.

[222] Kim Y.X., Steudle E. (2009). Gating of aquaporins by light and reactive oxygen species in leaf parenchyma cells of the midrib of *Zea mays*. J. Exp. Bot..

[223] Henzler T., Ye Q., Steudle E. (2004). Oxidative gating of water channels (aquaporins) in Chara by hydroxyl radicals. Plant Cell Environ..

[224] Yusupov M., Yan D., Cordeiro R.M., Bogaerts A. (2018). Atomic scale simulation of H_2_O_2_ permeation through aquaporin: Toward the understanding of plasma cancer treatment. J. Phys. D Appl. Phys..

[225] Wang Z., Zhao T., Hu Y., Zou L., Wang X., Zhang Y. (2021). Molecular dynamics simulations of the permeation and distribution of plasma ROS in aquaporin-1. Phys. Plasmas.

[226] Cui Y., Zhao T., Wang Z., Wang X., Wang D., Zhang Y. (2023). Molecular dynamics simulation of the effect of AQP1 on the transmembrane transport of plasma RONS across cancer cell membranes. Phys. Plasmas.

[227] Liu L., Liu R., Liu Y., Li G., Chen Q., Liu X., Ma S. (2021). Cystine-glutamate antiporter xCT as a therapeutic target for cancer. Cell Biochem. Funct..

[228] Lo M., Wang Y.Z., Gout P.W. (2008). The x cystine/glutamate antiporter: A potential target for therapy of cancer and other diseases. J. Cell. Physiol..

[229] Bannai S., Kitamura E. (1981). Role of proton dissociation in the transport of cystine and glutamate in human diploid fibroblasts in culture. J. Biol. Chem..

[230] Gout P., Kang Y., Buckley D., Bruchovsky N., Buckley A. (1997). Increased cystine uptake capability associated with malignant progression of Nb2 lymphoma cells. Leukemia.

[231] Jiménez-Vidal M., Gasol E., Zorzano A., Nunes V., Palacín M., Chillarón J. (2004). Thiol modification of cysteine 327 in the eighth transmembrane domain of the light subunit xCT of the heteromeric cystine/glutamate antiporter suggests close proximity to the substrate binding site/permeation pathway. J. Biol. Chem..

[232] Liu M., Tolg C., Turley E. (2019). Dissecting the dual nature of hyaluronan in the tumor microenvironment. Front. Immunol..

[233] Thapa R., Wilson G.D. (2016). The importance of CD44 as a stem cell biomarker and therapeutic target in cancer. Stem Cells Int..

[234] Misra S., Hascall V., Markwald R., Ghatak S. (2015). Interactions between hyaluronan and its receptors (CD44, RHAMM) regulate the activities of inflammation and cancer. Front. Immunol..

[235] Lin A., Truong B., Patel S., Kaushik N., Choi E.H., Fridman G., Fridman A., Miller V. (2017). Nanosecond-pulsed DBD plasma-generated reactive oxygen species trigger immunogenic cell death in A549 lung carcinoma cells through intracellular oxidative stress. Int. J. Mol. Sci..

[236] Liu X., Pu Y., Cron K., Deng L., Kline J., Frazier W.A., Xu H., Peng H., Fu Y.-X., Xu M.M. (2015). CD47 blockade triggers T cell–mediated destruction of immunogenic tumors. Nat. Med..

[237] Matlung H.L., Szilagyi K., Barclay N.A., van den Berg T.K. (2017). The CD47-SIRPα signaling axis as an innate immune checkpoint in cancer. Immunol. Rev..

[238] Weiskopf K. (2017). Cancer immunotherapy targeting the CD47/SIRPα axis. Eur. J. Cancer.

[239] McCracken M.N., Cha A.C., Weissman I.L. (2015). Molecular Pathways: Activating T Cells after Cancer Cell Phagocytosis from Blockade of CD47 “Don’t Eat Me” Signalsα-CD47–Mediated Cancer Cell Phagocytosis Activates T Cells. Clin. Cancer Res..

[240] Giannoni E., Fiaschi T., Ramponi G., Chiarugi P. (2009). Redox regulation of anoikis resistance of metastatic prostate cancer cells: Key role for Src and EGFR-mediated pro-survival signals. Oncogene.

[241] Kalay Z., Cevher S.C. (2012). Oxidant and antioxidant events during epidermal growth factor therapy to cutaneous wound healing in rats. Int. Wound J..

[242] Wee P., Wang Z. (2017). Epidermal growth factor receptor cell proliferation signaling pathways. Cancers.

[243] Jorissen R.N., Walker F., Pouliot N., Garrett T.P., Ward C.W., Burgess A.W. (2003). Epidermal growth factor receptor: Mechanisms of activation and signalling. EGF Recept. Fam..

[244] Iqbal N., Iqbal N. (2014). Human epidermal growth factor receptor 2 (HER2) in cancers: Overexpression and therapeutic implications. Mol. Biol. Int..

[245] Carpenter G., Cohen S. (1979). Epidermal growth factor. Annu. Rev. Biochem..

[246] Herbst R.S. (2004). Review of epidermal growth factor receptor biology. Int. J. Radiat. Oncol. Biol. Phys..

[247] Berlanga-Acosta J., Gavilondo-Cowley J., López-Saura P., González-López T., Castro-Santana M.D., López-Mola E., Guillén-Nieto G., Herrera-Martinez L. (2009). Epidermal growth factor in clinical practice–a review of its biological actions, clinical indications and safety implications. Int. Wound J..

[248] Henson E.S., Gibson S.B. (2006). Surviving cell death through epidermal growth factor (EGF) signal transduction pathways: Implications for cancer therapy. Cell. Signal..

[249] Stoscheck C.M., King Jr L.E. (1986). Role of epidermal growth factor in carcinogenesis. Cancer Res..

[250] Nasri Z., Memari S., Wenske S., Clemen R., Martens U., Delcea M., Bekeschus S., Weltmann K.D., von Woedtke T., Wende K. (2021). Singlet-Oxygen-Induced Phospholipase A(2) Inhibition: A Major Role for Interfacial Tryptophan Dioxidation. Chemistry.

[251] Lambeth J.D. (2004). NOX enzymes and the biology of reactive oxygen. Nat. Rev. Immunol..

[252] Sumimoto H., Miyano K., Takeya R. (2005). Molecular composition and regulation of the Nox family NAD (P) H oxidases. Biochem. Biophys. Res. Commun..

[253] Dutta S., Rittinger K. (2010). Regulation of NOXO1 activity through reversible interactions with p22phox and NOXA1. PLoS ONE.

[254] Shrestha P., Yun J.-h., Ko Y.-J., Kim M., Bae Y.S., Lee W. (2017). C-terminal tail of NADPH oxidase organizer 1 (Noxo1) mediates interaction with NADPH oxidase activator (Noxa1) in the NOX1 complex. Biochem. Biophys. Res. Commun..

[255] Skonieczna M., Hejmo T., Poterala-Hejmo A., Cieslar-Pobuda A., Buldak R.J. (2017). NADPH oxidases: Insights into selected functions and mechanisms of action in cancer and stem cells. Oxidative Med. Cell. Longev..

[256] Kamata T. (2009). Roles of Nox1 and other Nox isoforms in cancer development. Cancer Sci..

[257] Alekseeva A.S., Volynsky P.E., Krylov N.A., Chernikov V.P., Vodovozova E.L., Boldyrev I.A. (2021). Phospholipase A2 way to hydrolysis: Dint formation, hydrophobic mismatch, and lipid exclusion. Biochim. Biophys. Acta (BBA)-Biomembr..

[258] Han S.K., Kim K.P., Koduri R., Bittova L., Munoz N.M., Leff A.R., Wilton D.C., Gelb M.H., Cho W. (1999). Roles of Trp31 in high membrane binding and proinflammatory activity of human group V phospholipase A2. J. Biol. Chem..

[259] Stahelin R.V., Cho W. (2001). Differential Roles of Ionic, Aliphatic, and Aromatic Residues in Membrane−Protein Interactions: A Surface Plasmon Resonance Study on Phospholipases A2. Biochemistry.

[260] Gendaszewska-Darmach E. (2008). Lysophosphatidic acids, cyclic phosphatidic acids and autotaxin as promising targets in therapies of cancer and other diseases. Acta Biochim. Pol..

[261] Chen J., Ye L., Sun Y., Takada Y. (2017). A concise update on the relevance of secretory phospholipase A2 group IIA and its inhibitors with cancer. Med. Chem..

[262] Peng Z., Chang Y., Fan J., Ji W., Su C. (2021). Phospholipase A2 superfamily in cancer. Cancer Lett..

[263] Knowlden S.A., Hillman S.E., Chapman T.J., Patil R., Miller D.D., Tigyi G., Georas S.N. (2016). Novel inhibitory effect of a lysophosphatidic acid 2 agonist on allergen-driven airway inflammation. Am. J. Respir. Cell Mol. Biol..

[264] Jendzjowsky N.G., Roy A., Barioni N.O., Kelly M.M., Green F.H., Wyatt C.N., Pye R.L., Tenorio-Lopes L., Wilson R.J. (2018). Preventing acute asthmatic symptoms by targeting a neuronal mechanism involving carotid body lysophosphatidic acid receptors. Nat. Commun..

[265] Trotter A., Anstadt E., Clark R.B., Nichols F., Dwivedi A., Aung K., Cervantes J.L. (2019). The role of phospholipase A2 in multiple Sclerosis: A systematic review and meta-analysis. Mult. Scler. Relat. Disord..

[266] Junqueira R., Cordeiro Q., Meira-Lima I., Gattaz W.F., Vallada H. (2004). Allelic association analysis of phospholipase A2 genes with schizophrenia. Psychiatr. Genet..

[267] Smesny S., Kinder D., Willhardt I., Rosburg T., Lasch J., Berger G., Sauer H. (2005). Increased calcium-independent phospholipase A2 activity in first but not in multiepisode chronic schizophrenia. Biol. Psychiatry.

[268] Meng X., Kou C., Yu Q., Shi J., Yu Y. (2010). Schizophrenia: An association study targets phospholipase A2 genes as potential sites of susceptible genes. Psychiatry Res..

[269] Qasem H., Al-Ayadhi L., Al Dera H., El-Ansary A. (2017). Increase of cytosolic phospholipase A2 as hydrolytic enzyme of phospholipids and autism cognitive, social and sensory dysfunction severity. Lipids Health Dis..

[270] Meira-Lima I., Jardim D., Junqueira R., Ikenaga E., Vallada H. (2003). Allelic association study between phospholipase A2 genes and bipolar affective disorder. Bipolar Disord..

[271] Zanetti M.V., Machado-Vieira R., Joaquim H.P., Chaim T.M., Serpa M.H., de Sousa R.T., Gattaz W.F., Busatto G.F., Talib L.L. (2016). Distinct Glycogen Synthase Kinase 3 beta and Phospholipase A2 Expression Profiles in Bipolar I and II Disorders. Biol. Psychiatry.

[272] Leslie C.C. (2015). Cytosolic phospholipase A2: Physiological function and role in disease. J. Lipid Res..

[273] Chiarugi P., Pani G., Giannoni E., Taddei L., Colavitti R., Raugei G., Symons M., Borrello S., Galeotti T., Ramponi G. (2003). Reactive oxygen species as essential mediators of cell adhesion: The oxidative inhibition of a FAK tyrosine phosphatase is required for cell adhesion. J. Cell Biol..

[274] Wang W.Y., Pearson A.T., Kutys M.L., Choi C.K., Wozniak M.A., Baker B.M., Chen C.S. (2018). Extracellular matrix alignment dictates the organization of focal adhesions and directs uniaxial cell migration. APL Bioeng..

[275] Pirone D.M., Liu W.F., Ruiz S.A., Gao L., Raghavan S., Lemmon C.A., Romer L.H., Chen C.S. (2006). An inhibitory role for FAK in regulating proliferation: A link between limited adhesion and RhoA-ROCK signaling. J. Cell Biol..

[276] Lietha D., Cai X., Ceccarelli D.F., Li Y., Schaller M.D., Eck M.J. (2007). Structural basis for the autoinhibition of focal adhesion kinase. Cell.

[277] Calalb M.B., Polte T.R., Hanks S.K. (1995). Tyrosine phosphorylation of focal adhesion kinase at sites in the catalytic domain regulates kinase activity: A role for Src family kinases. Mol. Cell. Biol..

[278] Cohen L.A., Guan J.-L. (2005). Residues within the first subdomain of the FERM-like domain in focal adhesion kinase are important in its regulation. J. Biol. Chem..

[279] Grädler U., Bomke J., Musil D., Dresing V., Lehmann M., Hölzemann G., Greiner H., Esdar C., Krier M., Heinrich T. (2013). Fragment-based discovery of focal adhesion kinase inhibitors. Bioorganic Med. Chem. Lett..

[280] Kirchdoerfer R.N., Wang N., Pallesen J., Wrapp D., Turner H.L., Cottrell C.A., Corbett K.S., Graham B.S., McLellan J.S., Ward A.B. (2018). Stabilized coronavirus spikes are resistant to conformational changes induced by receptor recognition or proteolysis. Sci. Rep..

[281] Donoghue M., Hsieh F., Baronas E., Godbout K., Gosselin M., Stagliano N., Donovan M., Woolf B., Robison K., Jeyaseelan R. (2000). A novel angiotensin-converting enzyme–related carboxypeptidase (ACE2) converts angiotensin I to angiotensin 1-9. Circ. Res..

[282] Walls A.C., Park Y.-J., Tortorici M.A., Wall A., McGuire A.T., Veesler D. (2020). Structure, function, and antigenicity of the SARS-CoV-2 spike glycoprotein. Cell.

[283] Bunz O., Mese K., Funk C., Wulf M., Bailer S.M., Piwowarczyk A., Ehrhardt A. (2020). Cold atmospheric plasma as antiviral therapy–effect on human herpes simplex virus type 1. J. Gen. Virol..

[284] Chen Z., Garcia G., Arumugaswami V., Wirz R.E. (2020). Cold atmospheric plasma for SARS-CoV-2 inactivation. Phys. Fluids.

[285] Guo L., Yao Z., Yang L., Zhang H., Qi Y., Gou L., Xi W., Liu D., Zhang L., Cheng Y. (2021). Plasma-activated water: An alternative disinfectant for S protein inactivation to prevent SARS-CoV-2 infection. Chem. Eng. J..

[286] Ibrahim I.M., Abdelmalek D.H., Elshahat M.E., Elfiky A.A. (2020). COVID-19 spike-host cell receptor GRP78 binding site prediction. J. Infect..

[287] Ibrahim I.M., Abdelmalek D.H., Elfiky A.A. (2019). GRP78: A cell’s response to stress. Life Sci..

[288] Chu H., Chan C.-M., Zhang X., Wang Y., Yuan S., Zhou J., Au-Yeung R.K.-H., Sze K.-H., Yang D., Shuai H. (2018). Middle East respiratory syndrome coronavirus and bat coronavirus HKU9 both can utilize GRP78 for attachment onto host cells. J. Biol. Chem..

[289] Sabirli R., Koseler A., Goren T., Turkcuer I., Kurt O. (2021). High GRP78 levels in COVID-19 infection: A case-control study. Life Sci..

[290] Allam L., Ghrifi F., Mohammed H., El Hafidi N., El Jaoudi R., El Harti J., Lmimouni B., Belyamani L., Ibrahimi A. (2020). Targeting the GRP78-dependant SARS-CoV-2 cell entry by peptides and small molecules. Bioinform. Biol. Insights.

[291] Kumar N., Perez-Novo C., Shaw P., Logie E., Privat-Maldonado A., Dewilde S., Smits E., Berghe W.V., Bogaerts A. (2021). Physical plasma-derived oxidants sensitize pancreatic cancer cells to ferroptotic cell death. Free Radic. Biol. Med..

[292] Levine A.J. (1997). p53, the cellular gatekeeper for growth and division. Cell.

[293] Hou H., Sun D., Zhang X. (2019). The role of MDM2 amplification and overexpression in therapeutic resistance of malignant tumors. Cancer Cell Int..

[294] Picksley S.M., Lane D.P. (1993). What the papers say: The p53-mdm2 autoregulatory feedback loop: A paradigm for the regulation of growth control by p53?. BioEssays.

[295] Vassilev L.T., Vu B.T., Graves B., Carvajal D., Podlaski F., Filipovic Z., Kong N., Kammlott U., Lukacs C., Klein C. (2004). In vivo activation of the p53 pathway by small-molecule antagonists of MDM2. Science.

[296] Ranjan A., Bera K., Iwakuma T. (2016). Murine double minute 2, a potential p53-independent regulator of liver cancer metastasis. Hepatoma Res..

[297] Bouska A., Eischen C.M. (2009). Murine double minute 2: p53-independent roads lead to genome instability or death. Trends Biochem. Sci..

[298] Suzuki A., Toi M., Yamamoto Y., Saji S., Muta M., Tominaga T. (1998). Role of MDM2 overexpression in doxorubicin resistance of breast carcinoma. Jpn. J. Cancer Res..

[299] Li S., Li X., Zhao H., Gao M., Wang F., Li W. (2015). Overexpression of microRNA-125a-3p effectively inhibits the cell growth and invasion of lung cancer cells by regulating the mouse double minute 2 homolog/p53 signaling pathway. Mol. Med. Rep..

[300] Mori D., Nakafusa Y., Miyazaki K., Tokunaga O. (2005). Differential expression of Janus kinase 3 (JAK3), matrix metalloproteinase 13 (MMP13), heat shock protein 60 (HSP60), and mouse double minute 2 (MDM2) in human colorectal cancer progression using human cancer cDNA microarrays. Pathol.-Res. Pract..

[301] Yueh T.-C., Hung Y.-W., Shih T.-C., Wu C.-N., Wang S.-C., Lai Y.-L., Hsu S.-W., Wu M.-H., Fu C.-K., Wang Y.-C. (2018). Contribution of murine double minute 2 genotypes to colorectal cancer risk in Taiwan. Cancer Genom. Proteom..

[302] Abdel-Fattah G., Yoffe B., Krishnan B., Khaoustov V., Itani K. (2000). MDM2/p53 protein expression in the development of colorectal adenocarcinoma. J. Gastrointest. Surg..

[303] Chene P. (2004). Inhibition of the p53-MDM2 interaction: Targeting a protein-protein interface. Mol. Cancer Res..

[304] Verma S., Grover S., Tyagi C., Goyal S., Jamal S., Singh A., Grover A. (2016). Hydrophobic interactions are a key to MDM2 inhibition by polyphenols as revealed by molecular dynamics simulations and MM/PBSA free energy calculations. PLoS ONE.

[305] Beckerson P., Wilson M.T., Svistunenko D.A., Reeder B.J. (2015). Cytoglobin ligand binding regulated by changing haem-co-ordination in response to intramolecular disulfide bond formation and lipid interaction. Biochem. J..

[306] Tsujino H., Yamashita T., Nose A., Kukino K., Sawai H., Shiro Y., Uno T. (2014). Disulfide bonds regulate binding of exogenous ligand to human cytoglobin. J. Inorg. Biochem..

[307] Zhou D., Hemann C., Boslett J., Luo A., Zweier J.L., Liu X. (2017). Oxygen binding and nitric oxide dioxygenase activity of cytoglobin are altered to different extents by cysteine modification. FEBS Open Bio.

[308] Barford D. (2004). The role of cysteine residues as redox-sensitive regulatory switches. Curr. Opin. Struct. Biol..

[309] Shivapurkar N., Stastny V., Okumura N., Girard L., Xie Y., Prinsen C., Thunnissen F.B., Wistuba I.I., Czerniak B., Frenkel E. (2008). Cytoglobin, the newest member of the globin family, functions as a tumor suppressor gene. Cancer Res..

[310] McRonald F.E., Risk J.M., Hodges N.J. (2012). Protection from intracellular oxidative stress by cytoglobin in normal and cancerous oesophageal cells. PLoS ONE.

[311] Fordel E., Thijs L., Moens L., Dewilde S. (2007). Neuroglobin and cytoglobin expression in mice: Evidence for a correlation with reactive oxygen species scavenging. FEBS J..

[312] Hodges N.J., Innocent N., Dhanda S., Graham M. (2008). Cellular protection from oxidative DNA damage by over-expression of the novel globin cytoglobin in vitro. Mutagenesis.

[313] Li D., Chen X.Q., Li W.-J., Yang Y.-H., Wang J.-Z., Yu A.C.H. (2007). Cytoglobin up-regulated by hydrogen peroxide plays a protective role in oxidative stress. Neurochem. Res..

[314] Feng Y., Wu M., Li S., He X., Tang J., Peng W., Zeng B., Deng C., Ren G., Xiang T. (2018). The epigenetically downregulated factor CYGB suppresses breast cancer through inhibition of glucose metabolism. J. Exp. Clin. Cancer Res..

[315] Fujita Y., Koinuma S., De Velasco M.A., Bolz J., Togashi Y., Terashima M., Hayashi H., Matsuo T., Nishio K. (2014). Melanoma transition is frequently accompanied by a loss of cytoglobin expression in melanocytes: A novel expression site of cytoglobin. PLoS ONE.

[316] Chakraborty S., John R., Nag A. (2014). Cytoglobin in tumor hypoxia: Novel insights into cancer suppression. Tumor Biol..

[317] Ursini F., Maiorino M. (2020). Lipid peroxidation and ferroptosis: The role of GSH and GPx4. Free Radic. Biol. Med..

[318] Forcina G.C., Dixon S.J. (2019). GPX4 at the crossroads of lipid homeostasis and ferroptosis. Proteomics.

[319] Furuta T., Shi L., Toyokuni S. (2018). Non-thermal plasma as a simple ferroptosis inducer in cancer cells: A possible role of ferritin. Pathol. Int. (Lett. Ed.).

[320] Chen X., Yu C., Kang R., Tang D. (2020). Iron metabolism in ferroptosis. Front. Cell Dev. Biol..

[321] Hassannia B., Vandenabeele P., Berghe T.V. (2019). Targeting ferroptosis to iron out cancer. Cancer Cell.

[322] Yagi-Utsumi M., Tanaka T., Otsubo Y., Yamashita A., Yoshimura S., Nishida M., Kato K. (2021). Cold Atmospheric Plasma Modification of Amyloid β. Int. J. Mol. Sci..

[323] Bayliss D., Walsh J.L., Shama G., Iza F., Kong M.G. (2009). Reduction and degradation of amyloid aggregates by a pulsed radio-frequency cold atmospheric plasma jet. New J. Phys..

[324] Raschetti R., Albanese E., Vanacore N., Maggini M. (2007). Cholinesterase inhibitors in mild cognitive impairment: A systematic review of randomised trials. PLoS Med..

[325] Hamley I.W. (2012). The amyloid beta peptide: A chemist’s perspective. Role in Alzheimer’s and fibrillization. Chem. Rev..

[326] Yankner B.A., Duffy L.K., Kirschner D.A. (1990). Neurotrophic and neurotoxic effects of amyloid β protein: Reversal by tachykinin neuropeptides. Science.

[327] Hardy J.A., Higgins G.A. (1992). Alzheimer’s disease: The amyloid cascade hypothesis. Science.

[328] Wulff H., Castle N.A., Pardo L.A. (2009). Voltage-gated potassium channels as therapeutic targets. Nat. Rev. Drug Discov..

[329] Arispe N., Diaz J.C., Simakova O. (2007). Aβ ion channels. Prospects for treating Alzheimer’s disease with Aβ channel blockers. Biochim. Biophys. Acta (BBA)-Biomembr..

[330] Demuro A., Parker I., Stutzmann G.E. (2010). Calcium signaling and amyloid toxicity in Alzheimer disease. J. Biol. Chem..

[331] Liu F.-F., Liu Z., Bai S., Dong X.-Y., Sun Y. (2012). Exploring the inter-molecular interactions in amyloid-β protofibril with molecular dynamics simulations and molecular mechanics Poisson-Boltzmann surface area free energy calculations. J. Chem. Phys..

[332] Lemkul J.A., Bevan D.R. (2010). Assessing the stability of Alzheimer’s amyloid protofibrils using molecular dynamics. J. Phys. Chem. B.

[333] Brown A.M., Lemkul J.A., Schaum N., Bevan D.R. (2014). Simulations of monomeric amyloid β-peptide (1–40) with varying solution conditions and oxidation state of Met35: Implications for aggregation. Arch. Biochem. Biophys..

[334] Hou L., Shao H., Zhang Y., Li H., Menon N.K., Neuhaus E.B., Brewer J.M., Byeon I.-J.L., Ray D.G., Vitek M.P. (2004). Solution NMR studies of the Aβ (1−40) and Aβ (1−42) peptides establish that the Met35 oxidation state affects the mechanism of amyloid formation. J. Am. Chem. Soc..

[335] Torres W., Lameda V., Olivar L.C., Navarro C., Fuenmayor J., Pérez A., Mindiola A., Rojas M., Martínez M.S., Velasco M. (2018). Bacteria in cancer therapy: Beyond immunostimulation. J. Cancer Metastasis Treat..

[336] Kramer M., Masner M., Ferreira F., Hoffman R. (2018). Bacterial therapy of cancer: Promises, limitations, and insights for future directions. Front. Microbiol..

[337] Zahaf N.-I., Schmidt G. (2017). Bacterial toxins for cancer therapy. Toxins.

[338] Vasan N., Baselga J., Hyman D.M. (2019). A view on drug resistance in cancer. Nature.

[339] Pellegrini A., Thomas U., Bramaz N., Klauser S., Hunziker P., Von Fellenberg R. (1997). Identification and isolation of a bactericidal domain in chicken egg white lysozyme. J. Appl. Microbiol..

[340] Zheng K., Lu M., Liu Y., Chen Q., Taccardi N., Hüser N., Boccaccini A.R. (2016). Monodispersed lysozyme-functionalized bioactive glass nanoparticles with antibacterial and anticancer activities. Biomed. Mater..

[341] Osserman E. (1976). Postulated relationships between lysozyme and immunoglobulins as mediators of macrophage and plasma cell functions. Adv. Pathobiol..

[342] Vilcacundo R., Méndez P., Reyes W., Romero H., Pinto A., Carrillo W. (2018). Antibacterial activity of hen egg white lysozyme denatured by thermal and chemical treatments. Sci. Pharm..

[343] Sava G., Benetti A., Ceschia V., Pacor S. (1989). Lysozyme and cancer: Role of exogenous lysozyme as anticancer agent. Anticancer. Res..

